# The Fungus Among Us: Innovations and Applications of Mycelium-Based Composites

**DOI:** 10.3390/jof11080549

**Published:** 2025-07-23

**Authors:** Zahra Parhizi, John Dearnaley, Kate Kauter, Deirdre Mikkelsen, Priya Pal, Tristan Shelley, Paulomi (Polly) Burey

**Affiliations:** 1 Centre for Future Materials (CFM), University of Southern Queensland, Toowoomba, QLD 4350, Australia; zahra.parhizi@unisq.edu.au (Z.P.); priya.pal@unisq.edu.au (P.P.); tristan.shelley@unisq.edu.au (T.S.); 2 School of Agriculture and Environmental Sciences, University of Southern Queensland, Toowoomba, QLD 4350, Australia; john.dearnaley@unisq.edu.au; 3 School of Health and Medical Sciences, University of Southern Queensland, Toowoomba, QLD 4350, Australia; kate.kauter@unisq.edu.au; 4 School of Agriculture and Food Sustainability, University of Queensland, St. Lucia, QLD 4072, Australia; d.mikkelsen@uq.edu.au

**Keywords:** fungal mycelium, biocomposite, sustainability, waste stream, MBC

## Abstract

Mycelium-based composites (MBCs) are an emerging category of cost-effective and environmentally sustainable materials that are attracting significant research and commercial interest across various industries, including construction, manufacturing, agriculture, and biomedicine. These materials harness the natural growth of fungi as a low-energy bio-fabrication method, converting abundant agricultural by-products and waste into sustainable alternatives to energy-intensive synthetic construction materials. Their affordability and eco-friendly characteristics make them attractive for both research and commercialisation. Currently, mycelium-based foams and sandwich composites are being actively developed for applications in construction. These materials offer exceptional thermal insulation, excellent acoustic absorption, and superior fire safety compared to conventional building materials like synthetic foams and engineered wood. As a result, MBCs show great potential for applications in thermal and acoustic insulation. However, their foam-like mechanical properties, high water absorption, and limited documentation of material properties restrict their use to non- or semi-structural roles, such as insulation, panelling, and furniture. This paper presents a comprehensive review of the fabrication process and the factors affecting the production and performance properties of MBCs. It addresses key elements such as fungal species selection, substrate choice, optimal growth conditions, dehydration methods, post-processing techniques, mechanical and physical properties, termite resistance, cost comparison, and life cycle assessment.

## 1. Introduction

Over the previous decade, the construction sector has faced considerable challenges. Producing traditional construction materials consumes large amounts of energy and natural resources while polluting air, land, and water [1,2]. The demand for essential building materials like cement, bricks, and timber has surged with the growing global population, making the supply difficult to maintain [1]. Traditional bricks, made from natural materials like silica, alumina, lime, iron oxide, and magnesium, are widely used, leading to resource depletion and sustainability concerns, posing significant challenges for future generations [3,4]. As global populations grow, yearly agricultural consumption rises, increasing byproducts like rice husks, cotton stalks, as well as straw. The majority of these secondary products are treated as residual and often discarded or incinerated, releasing CO_2_, particulate matter, and other greenhouse gases into the atmosphere [5,6]. Although some byproducts are used in fertilisers, livestock bedding, and low-quality building materials like bricks, green concrete, insulators, non-load-bearing particleboards, or fill material in road construction [6], much of their potential remains untapped.

The environmental risks of engineered materials made from depletable sources, like petroleum and natural gas, have driven interest in sustainable, biodegradable alternatives for various technological applications, particularly in the construction sector. Biocomposites, particularly those made from mycelium, are now being explored as suitable building and construction materials [7,8,9]. Mycelium based biocomposites (MBCs) have been attracting attention in academic and commercial circles because their key component, mycelium, the actively growing structure of fungi responsible for nutrient absorption and colonisation, grow using minimal energy, produce no waste, and have diverse applications [10,11]. Mycelium form an interconnected system of delicate, filamentous structures called hyphae, each just 1–30 micrometres wide, starting from a single spore or hyphal fragment, which bind organic matter like plant and animal waste [12]. Mycelial filaments exhibit a multilayered architecture with unique chemical compositions, including proteins, glucans, and chitin [13]. Mycelium naturally binds organic matter through a network of hyphae, using nutrients from the substrate to grow. In nature, this organic matter comes from the remains and waste products of plants and animals [14]. Waste streams including cellulose, tannin, cutin, and lignin, plus proteins, fats, and other carbohydrates [15] can also be transformed by mycelium into valuable materials, converting agro-industrial residues with low or negligible market value into useful substances [16].

Natural materials like straw, sawdust, woodchips, cotton, and rice husks are commonly used as natural bases for creating nanocellulose as well as MBCs [17,18,19]. As fungi grow on these substrates, their hyphae weave through cellulose, hemicellulose, and lignin-rich materials, up-taking nutrients and integrating bonds to form MBCs [20]. MBCs offer benefits in comparison to conventional synthetic materials: they are cost-effective, lightweight, energy-efficient, biodegradable, and have a low carbon footprint [13,21]. MBCs also outperform synthetic materials like MDF, polyurethane, and polystyrene in recycling efficiency, supporting a circular economy with reduced emissions and improved land use [22].

MBCs are being explored for broad applicability, such as packaging [23], industrial tools [24], furniture [25], paper [26], building materials, textile films [27], insulation [7,28], and sound-absorbing and flooring composites [29]. In the building and construction industry, sustainable material development is gaining momentum for the advancement of low-cost, environmentally friendly materials that reduce dependence on fossil fuels and promote sustainable practices [30]. However, since construction is sensitive to variations in product quality, new materials like MBCs must undergo comprehensive structural integrity evaluations. The properties of MBCs are influenced by variables including substrate composition, fungal strain, growth conditions, and processing approaches [12,17]. Understanding these factors can improve production, allowing MBCs to be tailored for specific uses in the construction sector. Numerous reviews already exist on the advancement and application of mineral-based composites [17,29,31,32,33].

With controlled processing methods like hot pressing and precise growth environments, MBCs can offer features such as fire resistance as well as thermoacoustic insulation [10,26]. Mycelium is also being used in bio-integrated architecture, including building envelopes, where its flexibility and fire resistance make it an excellent insulating material. Studies show that building envelopes composed of mycelium sheets have a thermal conductivity of 0.1 W/mK, comparable to autoclaved aerated concrete [34], enhancing their viability as construction materials.

Material driven design (MDD) focuses on understanding the unique technical properties of mycelium to optimise its applications [35]. Successful mycelium cultivation requires specific ambient parameters, such as controlled light, temperature, and humidity, to prevent contamination [34]. Recent research by Santosh et al. investigated the compressive strength of MBCs [36]. While it was shown that unadulterated mycelium blocks are suitable for use as non-structural walls owing to their lower compressive strength [36], Ghazvinian et al. (2019) investigated the compressive strength of MBCs made from sawdust and straw as substrates, finding they lacked the needed for load-bearing structures [37]. However, Blauwhoff reported that densification through heat and pressure can improve the mycelium blocks’ strength by releasing trapped air, making them more viable for structural use [38].

## 2. Methods

A structured literature review was conducted to identify and analyse peer-reviewed studies on MBCs. Relevant studies were sourced from the Scopus, Web of Science, and Google Scholar databases between November 2024 and April 2025, using the keywords: “mycelium-based composites”, “fungi”, and (“mechanical properties” or “applications” or “production” or “biodegradability”). Search strategies were adjusted as needed to suit each database, and only English-language publications were considered.

The review process involved two screening stages: an initial screening of titles and abstracts to determine relevance, followed by a full-text review of eligible articles. Data were extracted and categorised based on fungal species, substrate composition, functional properties (e.g., mechanical strength, water absorption, shrinkage, fire resistance), and sustainability considerations. The findings were synthesised to outline current research trends, technological developments, and identified knowledge gaps within the field of MBCs.

## 3. Structural Biology of Fungi

Unlike plants, which primarily rely on cellulose for structural support, fungi utilise chitin and chitosan, which are sustainable biopolymers. Chitin, also found in most insect and arthropod exoskeletons, is a polysaccharide with a linear structure, consisting of *N*-acetylglucosamine monomers [39]. Fungal mycelium, composed of dense and intricate hyphal filament networks, contain glucans, manno-proteins, chitosan, chitin, polyglucuronic acid, and small amounts of proteins and glycoproteins [40,41]. These constituents endow mycelium with structural and mechanical characteristics comparable to lignocellulosic materials like wood and cork [42].

Hyphae develop from a spore, propagating through cell wall growth at the hyphal tips. They consist of compartments separated by septa, which facilitate the efficient transport of nutrients, water, and micro-molecules. This structure provides both protection and mechanical strength to the mycelium [13,14,15,16,17,18,19,20,21,22,23,24,25,26,27,28,29,30,31,32,33,34,35,36,37,38,39,40,41,42,43,44]. Hyphal structures can be categorised into three main types: generative, skeletal, and binding types [45].

Mycelial networks are typically classified as monomitic, dimitic, or trimitic systems, each defined by their hyphal structures [26,46]. Monomitic systems comprise entirely generative filaments, while dimitic systems include generative and skeletal hyphae. Trimitic systems incorporate all three types of hyphae [47]. White rot fungi include both monomitic and trimitic species, known for their ability to produce enzymes that effectively break down tough plant materials like lignin [48,49,50]. Research by Bayer and McIntyre’s indicates that monomitic mycelial networks exhibit lower structural integrity than dimitic and trimitic systems [47,51]. For instance, the trimitic system in *Trametes versicolor* demonstrated tensile and flexural strength superior to that of the monomitic system of *Pleurotus ostreatus* when cultivated on rapeseed straw [26].

However, many studies fail to specify the fungal species used in composite production, thus hindering reproducibility due to the omission of mycelium network details [52,53,54,55].

## 4. Materials

### 4.1. Fungal Species

Various fungal inoculants influence the mechanical characteristics of the final MBC [13,55]. Each fungal species uniquely affects factors like yield, mycelial filament thickness, morphology, and surface texture [17,26,56]. Wood-rotting fungi have gained scientific interest for their role in wood decomposition and biotechnological applications [45,57]. Various fungal species are recognised for their medicinal value, providing bioactive compounds such as polysaccharides, peptides, and proteins [56,58].

Sydor et al. (2022) [15] noted that most MBC research focuses on white rot fungi, with *Ganoderma lucidum* and *Pleurotus ostreatus* frequently cited for MBC production from 2012 to 2022 (*n* > 40 publications). *Trametes versicolor* was also frequently utilised and cited, appearing in 10 publications from 2012–2022. All of these fungi are known to cause white rot [15]. According to Sharma et al. (2024) [59], *Ganoderma* species are favoured in MBCs due to their rapid growth and ability to thrive on organic waste substrates. Notably, *Ganoderma lucidum* exhibits high elasticity, making it particularly suitable for packaging and construction materials [59]. Furthermore, these species can produce a tightly woven mycelial mat [15]. However, *Ganoderma* spp. also present certain drawbacks, such as high moisture absorption tendency, limited tensile resistance, vulnerability to biological degradation, and the necessity to inactivate the fungal species [26].

**Figure 1:**
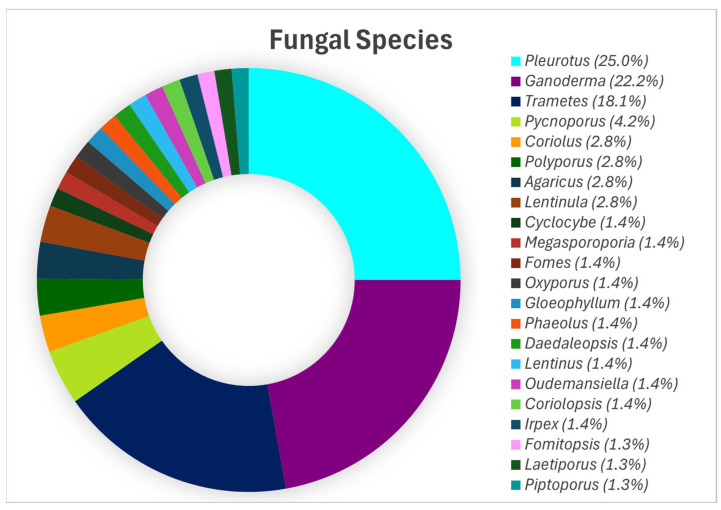
The primary fungal species utilised in the production of mycelium-based composites, reproduced under the Creative Commons Attribution License (CC BY 4.0) [60].

Aiduang et al. (2022) [60], after analysing 46 studies, reported that the genus *Pleurotus* leads in MBC production, accounting for 25.0% of total production, followed by *Ganoderma* (22.2%), *Trametes* (18.1%), and *Pycnoporus* (4.2%). Other contributors include *Polyporus*, *Agaricus*, *Coriolus*, and *Lentinula*, each at 2.8% (Figure 1).

### 4.2. Substrates

Agricultural waste streams consist primarily of lignocellulosic materials, including cellulose (35–50%), hemicelluloses (20–35%), and lignin (10–25%) [61,62]. These proportions vary depending on plant species, tissue type, and plant maturity. The global generation of agricultural waste has been increasing rapidly due to human activities, with a growth rate of 5–10% per year [63,64]. By 2025, global agro-industrial residues are projected to reach 2.2 billion tons annually [65,66]. Poor management of these residues poses environmental and health risks, including greenhouse gas emissions and water contamination, making agricultural waste a critical focus of scientific research [67].

Mycelium growth relies on substrates made from a blend of agricultural crop residues, which provide the necessary nutrients and conditions for fungal development [17,56]. Common substrates include rice husks, coconut husks, sawdust, and potato dextrose broth, all rich in cellulose [68], as well as banana fibres [69]. Rice bran, for example, has larger particles but retains more water due to its finer components [68]. Mycelium-based materials utilise lignocellulosic waste as a substrate, taking advantage of fungi’s natural ability to break down the cellulose and lignin found in plant biomass [60].

A comprehensive summary of research on MBC research from the past decade is presented in Table 1, with data collected using keywords from Web of Science and Google Scholar.

Haneef et al. [13] highlighted that a substrate combining refined cellulose and potato dextrose broth (PDB) in a 1:1 weight ratio is ideal for cultivating mycelium. Cellulose, abundant in hardwoods and crop residues, provides essential structural material, while PDB, abundant in simple sugars, is readily metabolised by mycelium as an energy source. This mixture creates a consistent substrate, enabling uniform mycelium growth and producing a homogenous material [13,126].

Since hyphae extract nutrients directly from the substrate, its composition significantly impacts mycelium growth [99]. Adding nutrient supplements can further promote growth, while fungal taxa and isolates vary in their capacity to degrade and colonise substrates based on lignocellulosic enzyme production [127]. Environmental parameters, including light, humidity, pH, temperature, and incubation duration also play key roles in mycelial growth, colonisation, and the structural properties of the final products [101,128,129].

Selecting the appropriate substrate is critical, as different fungal species thrive on specific materials, which directly affects composite development. The substrate not only supports fungal growth but also determines the mechanical characteristics of the final mycelium panel [89,130].

Mycelium forms a network that secretes enzymes to break down substrate polymers, converting them into the nutrients and minerals needed for growth. This process produces a compact fungal layer over the substrate, influencing the chemical, mechanical, and physical characterisations of macromycetes [17,128]. As the mycelium degrades and colonises the substrate, it uses the nutritional compound to extend and densify its hyphal network. For optimal growth, the substrate must provide carbon, nitrogen, minerals, vitamins, and water. Based on the fungal strains, the degradation process may preferentially target cellulose or lignin, although hemicellulose is commonly broken down by all species. These preferences are influenced by species-specific traits and environmental conditions [27,53,79].

### 4.3. Fungal Growth Conditions: Moisture Content and Temperature

Temperature and humidity significantly influence mycelium development. Optimal growth occurs at room temperature (24–25 °C) [131], with high humidity levels often maintained using humidifiers or sprinkler systems. Jiang et al. (2017) [55] used semi-permeable polypropylene bags to create sterile environments with up to 98% relative humidity, ideal for mycelium cultivation. Similarly, Attias et al. [24] incubated *Colorius* sp., *Trametes* sp., and *Ganoderma* sp. on woodchips at 23 °C and 95% relative humidity for a 14-day period prior to oven drying [24].

Naturally grown mycelium contains over 60% water [20], which must be reduced to halt growth and improve mechanical properties. While the specific final moisture content is underreported, it must be low enough to prevent fungal regrowth [56]. Moisture content varies by substrate and fungal species; for instance, hemp pulp retains more moisture than cotton wool [53]. Coatings also affect moisture absorption. Before deactivation, moisture content typically ranges from 59% [132] to 70–80% [133], while the final residual moisture is approximately 10–15% [133]. This residual moisture level is critical to the mechanical performance of an MBC.

### 4.4. Growth Profile and Biomass Fabrication

Fungal bio-composite production begins with substrate colonisation, which can be shaped either during or after mycelial growth. Once colonised, the material undergoes pressing and drying under controlled pressures and temperatures [134]. Most solid bio-composites use agricultural plant waste as a substrate, though one study used chicken feathers [135]. Forestry waste, including wood, fruit tree and bamboo fibres, is also common. Some patents propose wool and silk as alternative substrates [136].

Substrates must first absorb water to support fungal growth, with hydration times varying by material [20,137]. Although hydration time varies by substrate, pre-soaking for at least 48 h is generally required to achieve full water absorption and support fungal growth [138,139]. Once hydrated, raw materials are homogenised—via blending, grinding, or milling—to increase the surface area for fungal colonisation [20,82]. To prevent contamination, the substrate is sterilised before inoculation, usually via autoclaving, which maintains hydration. Alternative sterilisation methods include oven drying, which may excessively dehydrate the substrate, and hydrogen peroxide (H_2_O_2_) treatment, which is energy-efficient but more prone to contamination and less effective [140].

**Figure 2:**
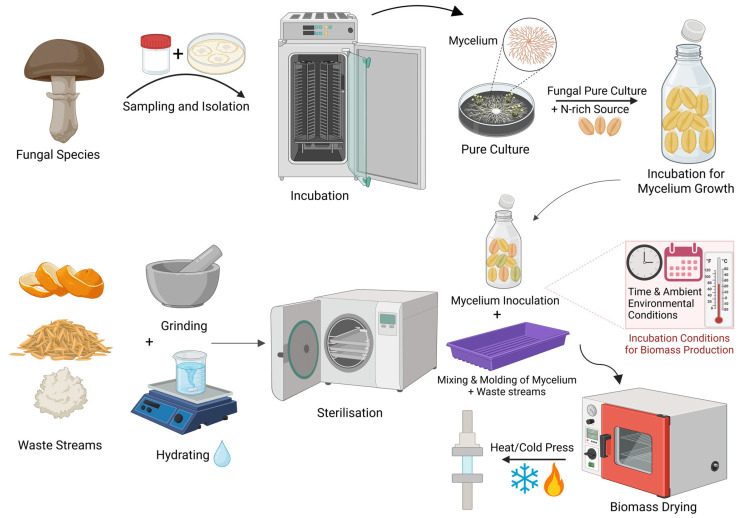
Schematic of the manufacturing process of mycelium-based composites. Created with BioRender.com.

After sterilisation, the substrate is mixed with fungal inoculum and placed in a mould. A 10-day incubation period allows mycelium facilitating substrate cohesion, forming a 3D network of fungal and plant fibres. Initially, the material contains about 70% water. Once moulded, it is oven-dried to stop mycelium growth. Water evaporation during drying creates microscopic air pockets, resulting in a rigid, closed-cell foam structure [80]. Figure 2 outlines the mycelium composite production cycle, highlighting key stages, their purpose, and process variations.

Fungal molding is used to create mycelium composites by shaping lignocellulosic materials into 3D moulds [10,141]. These materials are inoculated with 10–32 wt% fungal-derived biomass, including spores suspended in a fluid medium or hyphal/fruiting body tissues cultivated on the cultivation matrix enriched with nutrients like wheat grains [10,142]. Spores disperse uniformly, promoting even colonisation, though they initially struggle on low-quality materials. This limitation can be overcome by first growing on a nutrient-rich substrate like grain or sawdust before transitioning to lower-grade substrates, which results in fewer initiation points and uneven distribution [82].

After inoculation, moulds are incubated at room temperature or in controlled environments (25–27 °C) for periods ranging from days to months, contingent on the fungal strains, substrate, and desired material properties [143]. Room temperature incubation is more energy-efficient but slower than high-temperature conditions.

Post-incubation processes include heat-pressing [17,79] and integrating a composite woven-textile layer with a mycelium-derived foam core [84]. These methods stiffen the composite, halt fungal growth, and enhance mechanical performance [142]. In industrial settings, heat-pressing and oven drying are preferred for rapid dehydration and material densification. Another method was also employed for MBC preparation. In this approach, the mixture of substrate and fungal strains was placed into moulds and subjected to cold pressing. The moulds were then incubated for three weeks. After 21 days, the synthesised composites were removed and incubated outside the moulds for an additional week. The resulting specimens were thereafter oven-dried at 70 °C for 72 h [125].

**Figure 3:**
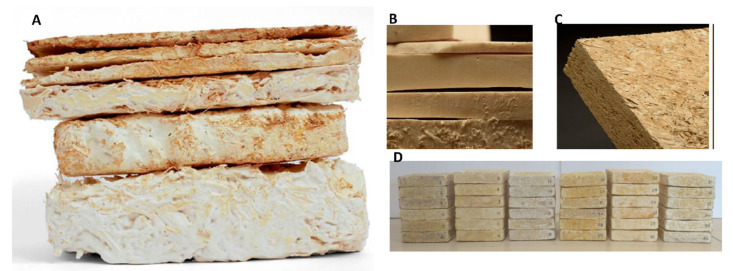
(**A**) The typical composition of mycelium-based foams, adapted under a Creative Commons Attribution-NonCommercial-NoDerivs 2.5 License [35]; In commercial applications, mycelium composites are used as: (**B**) substitutes for particleboard in wall panelling and door cores; (**C**) flexible insulation foams, under the terms of the Creative Commons CC BY license [26]; and (**D**) composites made by growing fungal mycelium on locally sourced vine pruning waste. The mycelium thoroughly colonises the plant substrate, resulting in a natural bio-composite foam, adapted with permission from [134]. There are no scale bars provided in the references.

Final mycelium composites are biodegradable, typically composed of ~95 wt% lignocellulosic material bound by ~5 wt% fungal mycelia (based on ergosterol concentrations of ~870 ppm, equating to 50 mg biomass per 1 g of wheat grains cultivated over seven days [144]. Adjusting water content during fabrication significantly impacts mechanical properties. Research by Appels et al. (2019) [17] highlights that pressing expels water and air, reducing porosity, increasing density, and improving Young’s modulus and strength [145,146,147]. Pressing also reorients fibres, enhances fibre connections, and minimises voids that could cause structural defects [148,149]. Hot pressing, which applies both pressure and heat, further strengthens the material compared to cold pressing [17]. Figure 3 presents various derivatives of MBC developed for different application areas.

## 5. Properties

Scientific studies evaluate mycelium composites through physical and mechanical tests, including density, compressive and flexural strength, heat resistance, water vapor transmission, moisture uptake, and dimensional stability [141]. Additional properties, such as acoustic insulation [79,150] and antibacterial benefits [13,53,76], are also documented. Table 2 compares the general characteristics of MBCs, bacterial cellulose (BC)-reinforced MBCs, and other materials.

### 5.1. Mechanical Properties

The mechanical properties of MBC are essential for engineering applications, with the fungal species and substrate significantly influencing their network structure and resulting strength variations [156].

#### 5.1.1. Tensile Strength

Tensile strength, a key performance metric of an MBC, ranges from 0.01 to 1.55 MPa (Table 3) and depends on the mycelium binder network [17,47,51,157]. Processing methods also impact tensile strength, particularly in construction applications [7]. For example, MBCs made with *T. versicolor* (trimitic hyphal system) on rapeseed straw exhibited greater tensile strength (0.04 MPa) than *P. ostreatus* (monomitic hyphal system) on the same substrate (0.01 MPa), due to the more complex, highly branched trimitic network of *T. versicolor* [17,157]. Pressing techniques further enhance tensile properties, with heat-pressing yielding the highest strength, succeeded by cold and/or non-pressing techniques [150,157,158]. A *P. ostreatus* composite cultivated on a cottonseed hull substrate reached 0.13 MPa with hot pressing (at 150 °C, 30 kN), compared to 0.03 MPa with cold pressing (20 °C for 20 min, followed by drying) [17]. MBCs’ tensile strength is comparable to that of polystyrene foam (0.15–0.7 MPa) [26].

#### 5.1.2. Compressive Strength

Compressive strength, a key mechanical characteristic, evaluates a material’s capacity to resist compressive loads and is crucial for functional applications [56]. Several factors influence the compressive strength of MBCs, including substrate formulation, fungal species, processing techniques, porosity, and pressing degree [17]. MBC compressive strength ranges from 0.03 to 4.44 MPa (Table 3), varying with substrate type [20,158,159,160].

Zimele et al. [18] assessed MBCs for building materials and found that hemp-based (0.36 MPa) and wood-based (0.52 MPa) MBCs exhibit compressive strengths comparable to cemented wood wool (0.3 MPa) and hemp concrete (0.36 MPa). MBCs made from pine sawdust with *Pycnoporus sanguineus* showed higher strength than those made with *Peniophora albidus* [89]. However, mycelium-based foam (MBF) from wheat stalks and *Pleurotus* species had a lower compressive strength than synthetic polymer foams due to higher water absorption [161]. Pultrusion has been suggested to improve the compressive strength of hemp-based MBCs [162].

Material composition also affects performance. Silverman (2018) [163] found that adding psyllium husk fibres enhanced MBF strength, while chicken feathers were also tested as reinforcements. MBCs made from *Ganoderma lucidum* cultivated over rapeseed cakes and oat husks outperformed those made from *Agaricus bisporus* and *Pleurotus ostreatus* on the same substrates [159]. *G. resinaceum* MBCs on rose flower waste (1.03 MPa) had greater compressive strength than those grown on lavender straw (0.72 MPa) [102]. Ghazvinian et al. [37] reported that a *P. ostreatus* MBC cultivated over sawdust (1.02 MPa) was significantly stronger than a *P. ostreatus* MBC colonised on straw (0.07 MPa). Additionally, *Trametes versicolor* MBC performed better when the fungus was grown on hemp rather than on pine or flax [20].

Increasing pressure during fabrication has been shown to improve compressive strength [158,164]. Ensuring adequate compressive strength is crucial for MBC applications in the packaging and building industries, as weaker materials pose structural limitations [7].

#### 5.1.3. Flexural Strength

Flexural strength, or the modulus of rupture or bend strength, measures the stress at which a material fractures under bending [157]. In MBCs, flexural strength is influenced by porosity (negatively), density (positively), and mycelium particle size [165]. Table 3 summarises the MBC flexural strength ranges from 0.05 to 4.40 MPa.

MBF generally has lower flexural strength than synthetic polymer foams and pulp fibre foams of similar density, though its tensile strength is significantly higher [161,166]. However, MBC made from *Trametes (T.) versicolor* and *Pleurotus (P.) ostreatus* on rapeseed straw and beech sawdust exhibited greater flexural strength than synthetic foams. *T. versicolor*, with its trimitic hyphal network, produced a stronger MBC (0.22 MPa) than *P. ostreatus*, which has a monomitic hyphal system (0.06 MPa) [17].

Substrate composition also plays a key role in MBC bending strength [167]. Fibrous straw-based composites outperformed cotton fibre composites, while beech sawdust composites had the highest flexural properties (flexural modulus: 9 MPa, flexural strength: 0.29 MPa) due to their dense mycelium network and continuous microstructure [80]. Incorporating 2.5% nanocellulose to a lignocellulosic substrate improved flexural strength from 1.5 to 3.5 MPa [86].

Jiang et al. (2017) [55] examined different fibre types in MBC and found that flax fibres provided better mycelium colonisation and bonding than jute or cellulose. Flax-based composites had nearly double the ultimate strength (35 kPa) and yield stress (27 kPa) of jute (20 kPa, 12 kPa) and cellulose (16 kPa, 15 kPa) [55].

**Table 3:** Mycelium-based composite’s mechanical properties based on substrate type.

Property	Substrate	Fungal Species	Value (MPa)
Compressive strength	Oat husk	*Agaricus bisporus*	0.06 [159]
		*Ganoderma lucidum*	0.13 [159]
		*Pleurotus ostreatus*	0.03 [159]
	Sawdust	*Ganoderma lucidum*	4.44 [158]
		*Ganoderma resinaceum*	1.32 [95]
		*Lentinus velutinus*	1.3 [89]
		*Pleutorus albidus*	0.4 [89]
		*Pleurotus ostreatus*	1.02 [37]
	Wheat straw	*Ganoderma lucidum*	0.07 [168]
		*Pleurotus* sp.	0.04 [161]
	MBC-Regardless of substrate (average)		0.36–0.52 [164] 0.17–1.1 [26]
Tensile strength	Rapeseed straw	*Pleurotus ostreatus*	0.1 [17]
		*Pleurotus ostreatus*	0.03 [17]
		*Pleurotus ostreatus*	0.24 [17]
		*Trametes versicolor*	0.04 [17]
		*Trametes versicolor*	0.15 [17]
	Sawdust	*Ganoderma lucidum*	1.55 [158]
		*Trametes versicolor*	0.05 [17]
	Wheat straw	*Pleurotus* sp.	0.05 [161]
	MBC-Regardless of substrate (average)		0.03–0.24 [7] Up to 0.343 [169]
Flexural strength	Rapeseed straw	*Pleurotus ostreatus*	0.06 [17]
		*Pleurotus ostreatus*	0.21 [17]
		*Trametes versicolor*	0.86 [17]
		*Trametes versicolor*	0.22 [17]
		*Pleurotus ostreatus*	0.87 [17]
	Sawdust	*Ganoderma lucidum*	2.68 [158]
		*Pleurotus ostreatus*	3.91 [25]
		*Trametes versicolor*	0.29 [17]
	Cotton	*Pleurotus ostreatus*	0.05 [17]
		*Pleurotus ostreatus*	0.24 [17]
		*Pleurotus ostreatus*	0.62 [17]
	BC-mycelium composite	*Trametes versicolor*	1.91–2.9 [151]
	MBC-Regardless of substrate (average)		0.87–15 [7] 0.05–0.29 [26]

### 5.2. Physical Properties

#### 5.2.1. Density

The density of the tested MBCs varies significantly across studies owing to variations in substrate type, fungal strains, and pressing methods [126,170]. Generally, higher density correlates with increased Young’s modulus and strength, as seen in the majority of porous materials [156].

Substrate composition plays a key role in determining an MBC’s density. Composites made from grain-, fibre-, husk-, or wood pulp-rich substrates tend to have higher densities [171,172]. Fungal species also influence density due to variations in lignocellulose degradation, which alters biomass composition [17,170]. For example, *A. bisporus*, *G. lucidum*, and *P. ostreatus* cultivated over rapeseed cake produced denser MBCs than those cultivated on oat husks [159]. Similarly, *Pycnoporus sanguineus* colonised on pine sawdust resulted in higher-density composites than those grown on coconut powder [89,173]. Table 4 summarises reported MBC densities ranging from 25 to 954 kg/m^3^.

Pressing techniques significantly increase final MBC density. Heat-pressing has been shown to triple density, while cold pressing doubles it, compared to non-pressed MBCs made from *P. ostreatus* and *T. versicolor* [17,20,158,174]. However, achieving consistent density and homogeneity in MBCs remains a challenge for large-scale applications [159,174].

**Table 4:** Mycelium-based composite’s density values based on substrate type.

Substrate	Fungal Species	Value (kg/m^3^)
Oat husk	*Agaricus bisporus*	36.0 [159]
	*Ganoderma lucidum*	25.0 [159]
	*Pleurotus ostreatus*	38.0 [159]
Sawdust	*Ganoderma lucidum*	130.0 [158]
	*Ganoderma lucidum*	954.0 [158]
	*Ganoderma resinaceum*	143.0 [95]
	*Trametes versicolor*	170.0 [17]
	*Trametes versicolor*	200.1 [175]
Pine sawdust	*Lentinus velutinus*	350.0 [89]
	*Pleutorus albidus*	300.0 [89]
	*Pycnoporus sanguineus*	320.0 [89]
Rapeseed cake	*Agaricus bisporus*	58.0 [159]
	*Ganoderma lucidum*	41.0 [159]
	*Pleurotus ostreatus*	49.0 [159]
Rapeseed straw	*Pleurotus ostreatus*	130.0 [17]
	*Pleurotus ostreatus*	240.0 [17]
	*Pleurotus ostreatus*	390.0 [17]
	*Trametes versicolor*	100.0 [17]
	*Trametes versicolor*	350.0 [17]
MBC-Regardless of substrate (average)		110–330 [150]

#### 5.2.2. Water Absorption Rate

MBCs are highly hygroscopic, meaning their water absorption rates are measured by comparing dry and post-moisture exposure weights [18]. This property is critical for structural applications, especially in construction [164]. Water absorption capacity is influenced by substrate density, with denser substrates generally absorbing less water [159]. This variation affects an MBC’s durability in moisture-exposed environments [20].

The fungal species and substrate type also influence water absorption. Substrates made from wood, coconut, and fibre materials typically retain more moisture [176]. For example, *T. versicolor* absorbs 26.8% and 30.3% water in wheat straw and flax, respectively, but 436% in rapeseed straw. *Ganoderma lucidum* on beech sawdust exhibits notably low absorption (6%) due to the hydrophobic characteristics of its hyphal walls, whereas *Ganoderma fornicatum* and *Ganoderma williamsianum* represent high water uptake in corn husk, rice straw, and sawdust [176]. Furthermore, Stratong-on et al. investigated the water absorption properties of composites made from *Pleurotus pulmonarius* (PP) and *Pleurotus ostreatus* (PO) both cultivated on sawdust. Their findings revealed that the PP-based composite exhibited an average mass increase of 203.44 ± 11.49% upon water absorption, whereas the PO-based composite showed a lower mass gain of 144.04 ± 13.89%, approximately 1.41 times less than that of the PP composite [177].

Water absorption is also affected by the mycelium’s outer hydrophobic layer. Higher-density mycelium layers reduce absorption, as seen in MBCs made from hemp substrates when compared to those made on flax and straw [20]. Similarly, MBCs made from *G. resinaceum* and rose flower waste (density: 462 kg/m^3^) absorbed less water (43.9%) than those made from lavender straw (114.6%, density: 347 kg/m^3^) [102]. *Pleurotus ostreatus* MBCs based on sawdust (330 kg/m^3^) absorbed less water than those on sugarcane bagasse (110 kg/m^3^) [91]. Smaller substrate particles reduce absorption by increasing density and minimising voids [17].

Compared to polymer-based materials (0.01 to 9%) [26,178], MBCs absorb significantly more water due to their cellulosic fillers and porous mycelium binder [179,180,181]. This remains a significant challenge for MBC applications in humid environments [7]. Strategies to mitigate water absorption include pressing techniques, granular fillers, and bio-derived coatings. Polyfurfuryl alcohol resin (PFA) has shown potential for improving water resistance in organic fibre composites [182], and chitosan coatings significantly reduce water uptake compared to carrageenan and xanthan coatings [161,183].

**Figure 4:**
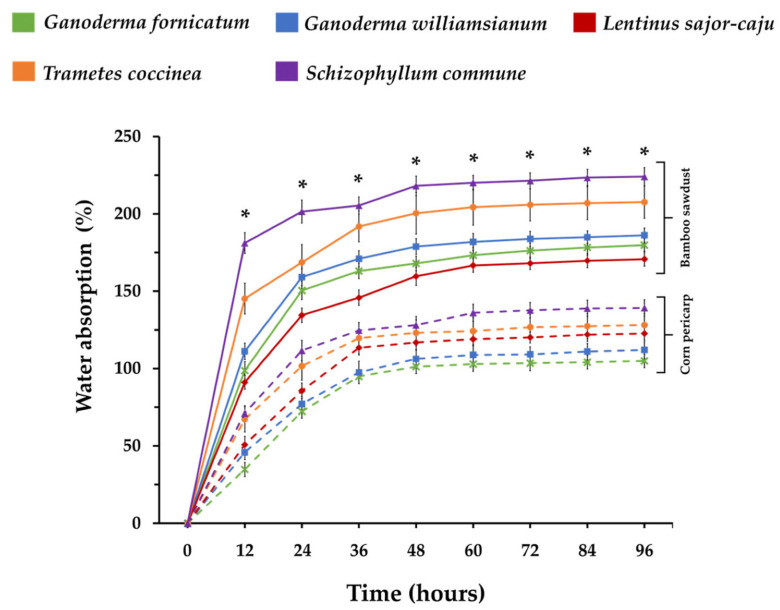
The water absorption abilities of mycelium-based materials from bamboo sawdust and corn peri-carp. “*” indicates a statistically significant difference at each point, as determined by Duncan’s multiple range test (*p* ≤ 0.05) within each substrate type in the experiment. Solid lines represent MBCs cultivated on bamboo sawdust, and dashed lines represent those grown on corn pericarp. Adapted under the Creative Commons Attribution License (CC BY 4.0) [121].

In one study, MBCs made from bamboo sawdust and corn pericarp were submerged in water for 96 h [121]. As can be seen from Figure 4, the bamboo MBC absorbed 170.70–224.08% water, stabilising after 48 h, while corn pericarp MBC absorbed 104.89% to 139.22%, stabilising at 60 h. Among bamboo composites, *Schizophyllum commune* had the highest absorption, while *Lentinus sajor-caju* had the least. Within corn pericarp composites, *G. fornicatum* absorbed the least water (Figure 4) [121].

#### 5.2.3. Acoustic Absorption Behaviour

MBCs are highly effective at absorbing sound, converting air molecule vibrations into heat and reducing noise buildup in enclosed spaces [26]. Some MBCs, such as those colonised on rice straw (52 dBa), hemp pith (53 dBa), and flax shive (53.5 dBa), outperform traditional sound absorbers like commercial ceiling tiles (61 dBa), urethane foam board (64 dBa), and plywood (65 dBa) [26].

Acoustic performance in MBCs is influenced by porosity, tortuosity, flow resistivity, and pressing conditions [79]. Pelletier et al. (2013) [79] found that MBCs made from cotton bur fibre, flax shive, hemp pith, kenaf fibre, rice straw, sorghum fibre, and switchgrass achieved 70–75% sound absorption at 1000 Hz. This makes them competitive alternatives to fibre boards (11–31%), polystyrene foams (20–60%), polyurethane foams (20–80%), plywood (10–23%), and softwood (5–15%) [26,184].

**Figure 5:**
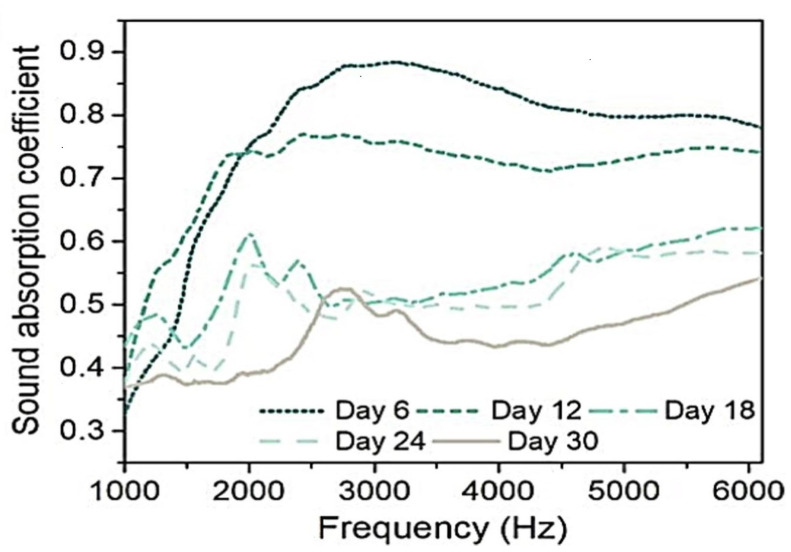
Sound absorption coefficient of mycelium-based foams in a 30 days incubation period, adapted with permission from [185].

A 2022 study examined the acoustic characteristics of a *T. versicolor* MBC made with yellow birch wood particles [185]. The maximum sound absorption coefficients exceeded 0.5 Hz across all samples, with the highest value (0.87 at 2800 Hz) observed in composites incubated for six days (Figure 5). Longer incubation periods led to increased porosity but reduced sound absorption. This effect was linked to mycelium growth gradually filling air gaps between wood particles, altering airflow resistance and reducing pore sizes, which affected sound transmission (Figure 5) [97]. Moreover, Walter and Gursoy studied MBCs produced by cultivating *Pleurotus ostreatus* on a substrate composed of shredded cardboard, newsprint, and paper. Their findings revealed that the composites exhibited the highest sound absorption in the high-frequency range, specifically between 2 kHz and 6.4 kHz [107].

Due to their strong acoustic absorption, MBCs serve as sound-insulating materials in the walls, doors, and ceilings of concrete halls and broadcasting studios [26,184]. However, pressing methods (hot or cold) can reduce sound absorption efficiency, making them unsuitable for MBCs intended as sound absorbers [186].

#### 5.2.4. Thermal Conductivity/Degradation

MBCs are effective natural thermal insulators because of their poor heat transfer properties, high porosity, low density, and significant air content [174,187]. Their thermal conductivity varies based on density, moisture content, and fibre type [26], ranging from 0.05 to 0.07 W/m·K—equal to traditional insulation materials like glass wool (0.04 W/m·K), extruded polystyrene (0.03 W/m·K), sheep wool (0.05 W/m·K), and kenaf (0.04 W/m·K) [23,188]. MBCs made with wheat straw and various mycelium species demonstrated heat transfer properties between 0.074 and 0.087 W/m·K, reinforcing their potential as sustainable insulators [83]. Sustainable composite insulators also contribute to reducing buildings’ environmental impact [189]. Dias et al. (2021) [189] examined a self-growing biocomposite made from *Miscanthus* × *giganteus* and mycelium, finding thermal conductivities between 0.0882 and 0.104 W/m·K, similar to straw (0.08 W/m·K) [190], hemp concrete (0.1 W/m·K) [187], softwoods (0.12 W/m·K) [191], biochar-doped wheat gluten (0.096 W/m·K) [192], and gypsum (0.17 W/m·K) [191].

MBCs undergo thermal degradation in three stages: initial water evaporation (25–200 °C, 5% weight loss), major degradation (200–375 °C, ~70% weight loss), and decomposition starting at 280–290 °C [193]. Their degradation range (225–375 °C) aligns with lignocellulosic materials (220–450 °C) [25,89,170,193,194]. Adding silica (SiO_2_) and glass fines significantly improves an MBC’s thermal resistance and fire-retardant properties [82]. Glass fines, in particular, extended flashover time from 94 to 370 s in wheat grain-based composites and from 75 to 311 s for rice hull-based composites [82,193]. Furthermore, furfurylation (treatment with furfuryl alcohol) reduced the fire growth rate index of wood-based MBCs from 15.17 to 1.99 (kW/m^2^ s) [195].

**Figure 6:**
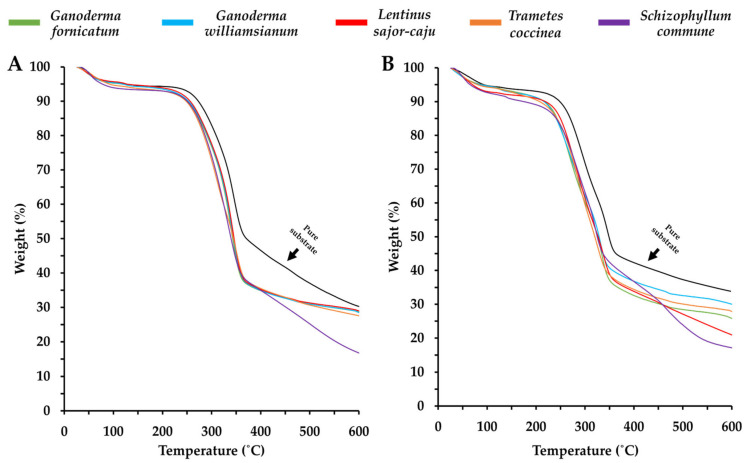
Thermogravimetric analysis (TGA) on the MBC produced, using a combination of each fungal species with either bamboo sawdust (**A**) or corn pericarp (**B**), reproduced under the Creative Commons Attribution License (CC BY 4.0) [121].

Mycelium exhibits better fire resistance than thermoplastics like polymethyl methacrylate (PMMA) and polylactic acid (PLA) due to its higher char yield [193,196]. It improves the fire resistance of wheat grain composites [82], though extending its growth period beyond six days has minimal effect on fire properties [193]. The thermal degradation behaviour of mycelium varies with temperature because of simultaneous chemical and thermal processes. Initially, up to 100 °C, no heat is released, indicating a non-combustion phase driven by water loss [193,194]. Between 100 °C and 200 °C, heat release increases as flammable volatiles are emitted [193]. Thermogravimetric analysis (TGA) of MBCs (Figure 6) [121] confirms a three-phase degradation pattern, similar to lignocellulosic substrates but with a more rapid weight loss rate [17,89]. The pure substrates—bamboo sawdust and corn pericarp—exhibited slower weight loss compared to the fungal-colonised samples, suggesting that fungal colonisation increases the substrate’s sensitivity to thermal degradation, which may reduce the thermal stability, durability, and structural strength of MBCs [17,121]. However, the degradation of the composites shown in Figure 6 occurs within the temperature range of synthetic foams (250–475 °C) and several paper-based materials (250–350 °C), highlighting the potential of MBCs for a broad scope of insulation applications [111,197,198].

#### 5.2.5. Shrinkage

Shrinkage is a key physical characteristic of MBCs, primarily caused by dehydration during drying [20]. Lower shrinkage improves strength and shape stability. A 2024 study found that MBCs made from bamboo sawdust had lower shrinkage (3.14% to 5.83%) than those made from corn pericarp (9.80% to 16.66%) across various fungal species [121]. *L. sajor-caju* on bamboo sawdust MBC showed the least shrinkage, while *S. commune* on corn pericarp MBC showed the highest [121]. Moisture content and drying methods also influenced shrinkage [17,20].

**Figure 7:**
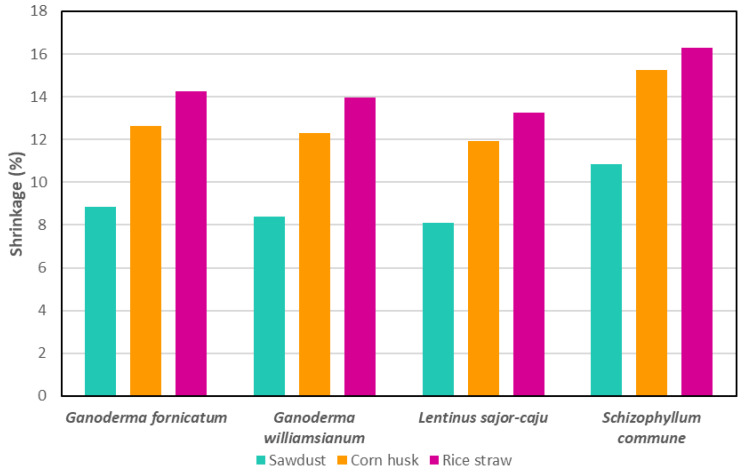
Shrinkage percentage for different fungal species grown on rice straw, corn husk and sawdust. Data adapted from [111].

These findings align with previous studies, which reported MBC shrinkages between 2.78% and 17% [111,141,199,200,201]. Notably, MBCs using bamboo sawdust consistently showed lower shrinkage, highlighting the role of substrate selection in minimising shrinkage [20,141]. In another study, MBCs made from rice straw had the maximum shrinkage, followed by corn husk and sawdust, regardless of fungal species (Figure 7) [111]. *S. commune* consistently had the highest shrinkage across substrates, while *L. sajor-caju* had the lowest, though its shrinkage was not significantly different from those of *Ganoderma fornicatum* and *Ganoderma williamsianum*. These results suggest potential for MBCs as alternatives to wood insulation boards [111].

MBCs made from *Pleurotus* sp. on wheat residue had a shrinkage value of 6.2% [141], while Elsacker et al. (2019) [20] reported higher shrinkage for *T. versicolor* MBCs on pine softwood waste (15%), flax (10%), and hemp (9%), emphasising the impact of substrate choice. Compared to polymer-based materials like nylon, polystyrene, and polypropylene (0.3% to 2.5% shrinkage) [202], MBCs exhibit a broader shrinkage range, similar to wood-based materials (1% to 25%) [203,204].

## 6. Scanning Electron Microscopy Analysis

Mycelium composites exhibit complex surface topography, best analysed using Scanning Electron Microscopy (SEM) to examine their morphology and structural characteristics [205].

**Figure 8:**
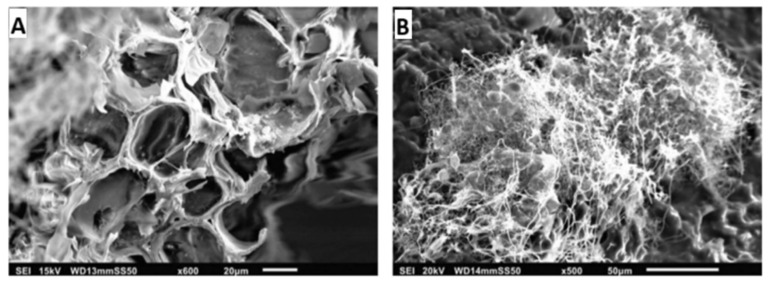
SEM image of: (**A**) fibre structure of *Miscanthus*; and (**B**) the mycelium network within the *Ganoderma resinaceum*, reproduced under the terms of the Creative Commons CC BY license [189].

A study on mycelium-*Miscanthus* composites (sample G0.7_M1_P0.5) utilised SEM to analyse *Ganoderma resinaceum* mycelium, *Miscanthus* fibres, and potato starch using SEM [189]. *Miscanthus* fibres displayed an anisotropic structure with aligned hollow tubes, while mycelium formed an interconnected filament network. In the composite, mycelium enveloped the *Miscanthus* internally and externally, though the penetration depth remained unclear. Voids observed in the SEM images suggested potential variations in mechanical properties (Figure 8) [189].

**Figure 9:**
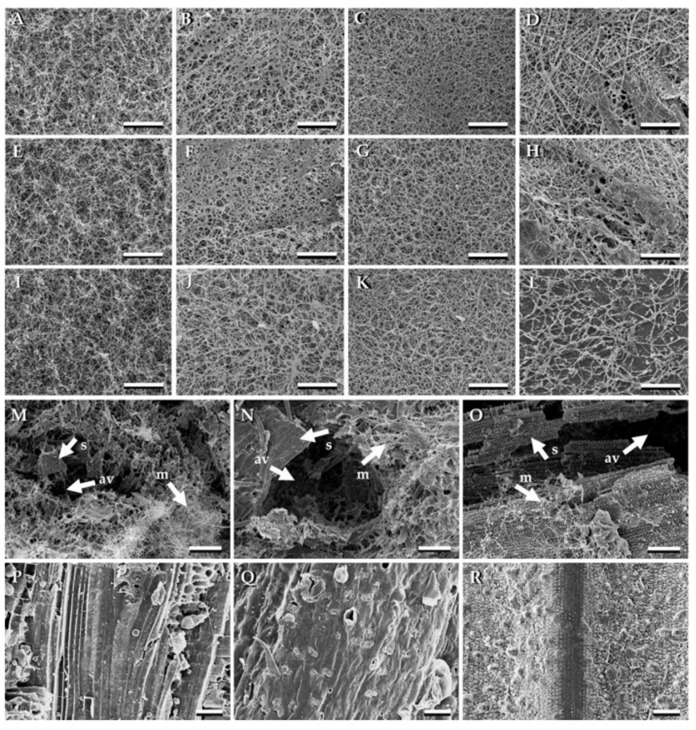
Scanning electron microscopy (SEM) images of mycelium-based composites derived from different fungal species and substrates. The MBC surfaces synthesised from *Ganoderma fornicatum* with sawdust (**A**); corn husk (**E**); and rice straw (**I**). The MBC surfaces synthesised from *Ganoderma williamsianum* with sawdust (**B**); corn husk (**F**); and rice straw (**J**). The MBC surfaces synthesised from *Lentinus sajor-caju* with sawdust (**C**); corn husk (**G**); and rice straw (**K**). The MBC surfaces synthesised from *Schizophyllum commune* with sawdust (**D**); corn husk (**H**); and rice straw (**L**). The cross sections of MBC synthesised from *Lentinus sajor-caju* with sawdust (**M**); corn husk (**N**); and rice straw (**O**). The uncolonised sawdust (**P**); corn husk (**Q**); and rice straw (**R**). Arrows indicated substrate (s), fungal mycelia (m), and air-voids (av). Scale bar; (**A**–**O**) = 100 µm and (**P**–**R**) = 50 µm, reproduced under the Creative Commons Attribution License (CC BY 4.0) [111].

SEM analysis of MBCs revealed that fungal mycelia uniformly covered all composite surfaces (Figure 9A–L). *L. sajor-caju* exhibited higher mycelial density across various substrates. Cross-sectional images showed mycelial filaments interconnecting substrate particles, with trapped air pockets within the composites (Figure 9M–O) [111]. These findings align with previous studies [17,170]. In contrast, uninoculated substrates lacked both fungal mycelium and trapped air pockets (Figure 9P–R) [111].

Islam et al. (2017) [12] used SEM to analyse fibre arrangement, revealing an irregular microstructural network of randomly oriented fibres. The average hyphae diameter was 1.3 ± 0.66 µm [12].

## 7. Fourier Transform Infrared (FTIR) Spectroscopy

**Figure 10:**
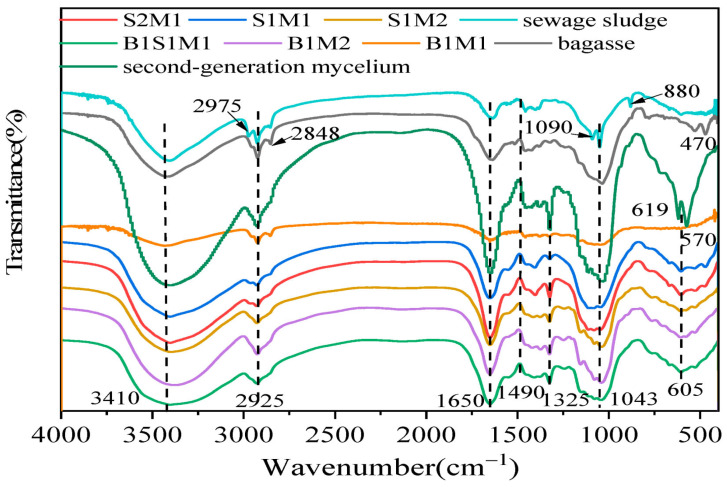
FTIR spectra of sewage sludge (SM), bagasse (BM), and a mixture of bagasse plus sewage sludge (BSM) as substrates and manufactured MBC using *Pleurotus ostreatus.* Sample labels represent different substrate-to-mycelium mass ratios. B1M2, B1M1, and B2M1 refer to bagasse–ready-made mycelium composites with mass ratios of 1:2, 1:1, and 2:1, respectively. B1S1M1 corresponds to a composite made from bagasse-sewage sludge-ready-made mycelium in a 1:1:1 ratio. Similarly, S1M2, S1M1, and S2M1 denote sewage sludge–ready-made mycelium composites with mass ratios of 1:2, 1:1, and 2:1, respectively. Reproduced under the Creative Commons Attribution License (CC BY 4.0) [206].

FTIR spectroscopy is commonly used to gather information about the chemical and structural characteristics of MBCs. The resulting spectra also provide valuable insights into the functional groups and molecular identifications of the substrates and final composites. In a recent study by Hu and Cao, FTIR was employed to analyse the chemical composition of the substrates and manufactured panels [206]. As illustrated in Figure 10, FTIR spectra display characteristic peaks and bands corresponding to various functional groups. For example, a stretching around 3410 cm^−1^ is attributed to O–H stretching vibrations of polysaccharides, indicating the presence of cellulose and hemicellulose [56]. Absorption bands observed at 1650 cm^−1^ are associated with C=O stretching (amide I) and NH_2_ groups [207], while the band at 1490 cm^−1^ corresponds to CH_2_ stretching vibrations, indicative of protein content. A notable band at 1325 cm^−1^ is linked to NH_2_ stretching in amines, commonly referred to as amide III [86]. Additionally, the peak detected at 1043 cm^−1^ is characteristic of C–C vibrations, suggesting the presence of proteins, lignin, and polysaccharides [206].

Another study by Haneef et al. investigated the FTIR spectra of MBCs derived from various fungal species, regardless of substrate type. They reported that *G. lucidum* exhibited a greater presence of lipids, while *Pleurotus ostreatus*, in contrast, displayed stronger spectral bands, likely originating from polysaccharides [13].

Overall, infrared spectroscopy of the mycelium composites highlighted distinct absorption patterns corresponding to their molecular constituents. These included lipids, indicated by absorption bands in the 3000–2800 cm^−1^ range and around 1737 cm^−1^ (associated with ester bonds); proteins, with characteristic amide I, II, and III bands observed between 1700–1300 cm^−1^; nucleic acids, detected around 1255–1245 cm^−1^; and polysaccharides, showing distinct signals in the 1200–900 cm^−1^ region [13,89,151].

## 8. Cost Comparison

The production costs of MBCs vary across industries, influenced by feedstock accessibility, manufacturing methods, labour costs, and industry dynamics [164,208]. MBCs can be cost-effective due to their reliance on agro-industrial byproducts, energy-efficient manufacturing, and lower ecological footprint. However, precise cost comparisons with traditional materials remain challenging [164].

Osman (2023) [209] estimated construction costs for various building materials (Table 5), showing mycelium-plywood panels as the most affordable and concrete blocks as the most expensive materials. Mycelium panel production is estimated at $0.83 per cubic foot [209]. Research indicates that MBCs are more cost-effective than one of their major competitors, expanded polystyrene (EPS) foam, which typically costs between $5 and $13 depending on its density [210,211]. Studies also indicate MBCs can reduce costs by over 65% compared to paper-derived materials and over 90% versus fabric composites, gypsum, polymers, and wood-PHA composites [164,212,213]. However, savings depend on application, production scale, and regional factors [214]. Additionally, MBC costs are comparable to cement-derived materials [215]. These economic advantages, mostly in material sourcing, production, and waste reduction, make MBCs a competitive alternative in various industries [209].

## 9. Termite Resistance

Termites cause extensive structural damage worldwide, amounting to billions of dollars annually [216]. While most prevalent in Africa, Asia, South America, and Australia, they also impact North America, with New Orleans alone experiencing over $100 million in damage each year just in New Orleans [217]. MBCs, composed mainly of lignocellulose, are naturally susceptible to termites. However, resistance can be enhanced by optimising substrate composition and applying natural or commercial termiticidal treatments [218,219].

Research shows termites predominantly degrade the base of MBC samples rather than the sides or top [218]. Termiticide efficacy correlates with termite mortality, with higher mortality indicating stronger repellence. Vetiver oil, cedar oil, and guayule resin exhibit varying degrees of repellence [218]. Among tested biocomposites, hemp-based MBCs demonstrated the highest resistance and lowest loss over four weeks (16–53 wt%), while kenaf-based MBCs showed medium to high resistance but higher mass loss (43–62 wt%), depending on the mycelium strains. Corn-based MBCs had lower termite resistance, moderate mortality rates, and mass loss of 42–43 wt% [218].

Guayule resin, rich in flavonoids, cinnamic compounds, terpenoids, and p-anisic acid [220], and vetiver oil, containing α- and β-vetivone compounds [221], are highly effective natural termiticides. A single-layer treatment with these oils ensures total termite mortality and significantly reduces mass loss in treated MBCs (18–28 wt% for guayule resin and 16–27 wt% for vetiver oil), compared to untreated MBCs (42–62 wt%) and untreated southern yellow pine (80 wt%) [221].

## 10. Life Cycle Assessment

MBCs are biodegradable, cost-effective, and grow on readily available substrates [4]. In contrast, traditional construction materials contribute to pollution by releasing harmful emissions during production [4]. This highlights the importance of evaluating materials through life cycle assessment (LCA), which analyses environmental impacts from production to disposal [56].

Challenges in MBC production include precision in drying, forming, and cutting, as identified in 2016 [208]. Durability is another concern, with studies suggesting mycelium bricks may last less than 50 years [222]. Research is needed to enhance longevity while maintaining biodegradability. Despite these challenges, MBCs exhibit substantially lower embodied energy compared to traditional materials—they are up to 80 times more sustainable than concrete [154]. Their eco-costs are also lower, as they utilise organic waste for production [4]. Table 6 compares the lifespan, fossil energy demand, climate impact, and eco-costs of various building materials.

**Figure 11:**
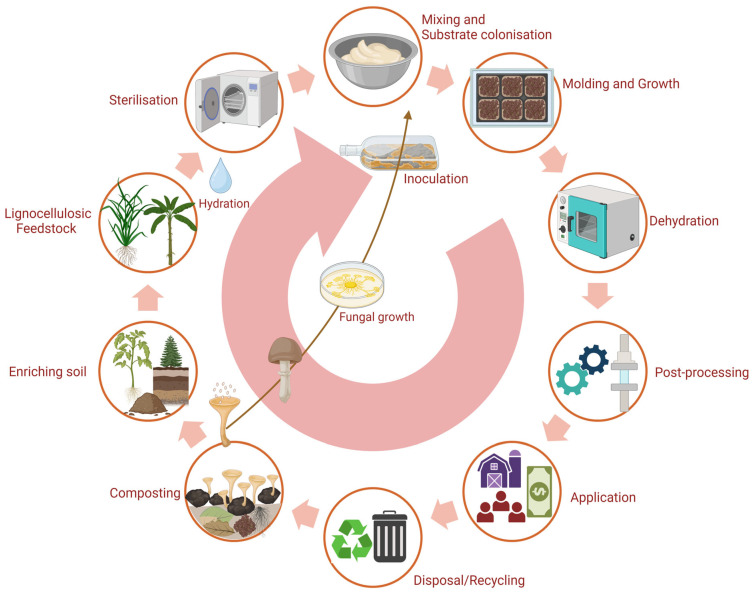
The life cycle of mycelium-based materials. Created with BioRender.com.

The manufacturing of MBCs has a lower ecological footprint than materials such as extruded polystyrene and rockwool [223]. However, its dependence on biogenic resources such as hemp and sawdust affects its properties. To minimise environmental impact, using locally sourced biogenic waste is recommended. While MBCs’ end-of-life impacts remain understudied, wood-fibre and straw panels currently have lower climate change impacts [223,225]. Nonetheless, MBCs require less fossil energy than any traditional insulation materials and hold potential for applications beyond construction, including packaging, furniture, and fashion [223]. Figure 11 illustrates the production and life cycle of MBCs.

Unlike some novel substitutes for traditional structural materials, such as fibre-reinforced polymer (FRP) bars, which require costly waste disposal methods or complex recycling processes [226], MBCs can be easily recycled. Alaux et al. (2024) [227] examined MBCs’ end-of-life scenarios, comparing incineration to partial recycling, where 70% of panel mass replaces beech sawdust in new product cycles. Industrial-scale production reduced most environmental impacts, including a 45% decrease in global warming potential (GWP), but increased terrestrial ecotoxicity. The main contributor to residual greenhouse gas (GHG) emissions was electricity consumed from mixed energy sources. Adjusting energy inputs in manufacturing could reduce GHG emissions by 64% [227]. Previous studies show that transitioning to renewable energy in production and supply chains can lower emissions for insulation materials by up to 83% [228,229,230].

## 11. Future Directions and Outlook

Fungi have long played a significant role in medicine, biotechnology, construction, and food production. Research into mycelium and its composite materials offers valuable insights into fungal network structures and their biological functions. Further studies can drive eco-friendly, lightweight, and mechanically robust composites [55,56,141]. This review integrates both experimental and simulation-based approaches to support these advancements. Recent research and commercialisation have highlighted the extensive potential of MBCs. They are being explored for use in packaging [21,141,231], thermal insulation [20,83,141,232], consumer electronics [233], acoustic absorption foams [79,184], and fire-resistant applications [82,234]. Additionally, they are being integrated into construction as panelling, flooring, and furnishings [235,236,237]. Their water absorption properties make them promising for superabsorbent materials [238,239,240], while their natural hydrophobicity suggests applications in coatings [13,82] and textiles [241,242]. Mushroom residues have recently been used in the production of cosmetic facial masks due to their antioxidative characteristics [243].

Mycelium also contains valuable biopolymers, including chitin, chitosan, and β-glucan, which can be extracted and used in 3D-printing, cellulose nanocomposites, films, sheets, and nano-papers [11,244,245]. This could lead to sustainable alternatives for synthetic polymers in filtration membranes [246,247], printed circuit boards [248], and sports equipment [135,249]. Several methods can be applied to improve the physical and mechanical properties of MBCs. Some of promising techniques that have been successfully applied to nanocomposites involve impregnating them with kombucha bacterial cellulose, resin, and nanofibrillar cellulose, leading to increased stiffness, tensile strength, and thermal conductivity [250,251,252]. A similar approach could be explored to improve MBCs. Additionally, advanced manufacturing techniques like extrusion and pultrusion could enhance the production of the bio-composites [253,254,255].

Beyond materials science, mycelium networks play a critical ecological role by facilitating communication and nutrient exchange among plants, supporting pest and disease resistance [256,257,258]. In sustainable agriculture, fungi function as biocontrol agents, promoting plant growth while reducing reliance on chemical pesticides, fungicides, and fertiliser [259]. Their use in microbial inoculants offers a cost-effective, eco-friendly alternative to chemical treatments [259,260].

Additionally, fungi can help degrade persistent organic pollutants due to their secretion of lignolytic extracellular enzymes and acidic metabolites [261]. Introducing fungal inoculants can enhance crop yields, soil health, and plant resilience against stressors like salinity, drought, and temperature fluctuations. As key components of the plant microbiome, fungi contribute to sustainable agriculture by supporting ecosystem balance and phytobiome engineering for improved crop production [262].

## 12. Conclusions

Mycelium cultivation offers an energy-efficient bio-fabrication approach for repurposing agricultural residues into eco-friendly substitutes for synthetic building materials. These include acoustic and thermal insulation, door cores, panelling, flooring, cabinetry, and furnishings. Different applications require tailored properties: high porosity and low density for acoustic and insulation, and scratch resistance, flexural strength, and stiffness for structural components. The environmental benefits and versatility of MBCs make them increasingly sought after.

This review examines MBC fabrication, physical and mechanical properties, cost-effectiveness, and life cycle assessments. It highlights key bio-fabrication factors, including fungal species, substrate types, and environmental conditions (temperature, moisture, aeration) and their influence on the material properties. MBCs excel in thermo-acoustic insulation, with thermal conductivities equal to or lower than commercial insulators and superior sound absorption compared to ceiling tiles, polyurethane foams, and plywood. Moreover, these composites offer enhanced fire resistance over traditional materials like extruded polystyrene and particleboard, as well as natural termite resistance.

Despite these advantages—low cost, biodegradability, safety, and minimal environmental impact—MBCs face challenges, including low mechanical strength, weathering susceptibility, scalability issues, limited lifespan (<50 years), and a lack of standardised manufacturing/testing methods. Overcoming these obstacles is essential for broader adoption. This review provides a comprehensive resource for researchers entering the field, offering insights into MBC production and potential applications.

## Figures and Tables

**Table 1:** A summary of research on mycelium-based materials over the past decade (2014–present), using data from Web of Science and Google Scholar.

Fungal Species	Substrate Type	Application	Year	Reference
N/A	woven textile and natural glue (water, starch, maltodextrin), kenaf pith	shoe sole	2014	[70]
N/A	core: kenaf and hemp. Textile skins: Biotex jute, flax, Biomid cellulose fibre	structure, construction	2014	[71]
*Oyster mushroom*	cotton seed hulls, carboxylated styrene butadiene rubber (sbr) latex, Silane coupling agent	structure, construction	2014	[72]
*G. lucidum*	N/A	sandwich composites	2014	[73]
N/A	ground corn stover, reinforcement layers: jute textile, kenaf mat, glue: G242 industrial corn starch, maltodextrin glue	shoe sole, integral tooling	2014	[70]
N/A	N/A	insulation panels	2015	[74]
*C. versicolor*, *P. ostreatus*	wood chips, hemp hurd, loose hemp fibre and nonwoven, mats of hemp fibre	insulating foam	2015	[75]
N/A	core: cotton (ginning waste), hemp shell: woven or nonwoven mat	packaging	2016	[53]
*P. ostreatus*	wood sawdust	Structure, construction	2016	[53]
N/A	Ecovative DIY and psyllium, chia and linum seeds	N/A	2016	[52]
*G. lucidum*	wood, additives	subtractive manufacture	2016	[76]
*L. edodes, P. ostreatus,* *G. lucidum*	wood shavings, straw, corn stalk and rice husks	Structural furniture	2016	[77]
*Pleurotus* sp.	wheat residues (*Triticum* sp.)	food & packaging	2016	[77]
N/A	core: corn stover, hemp; Shell: (a) Biotex Jute, (b) Biotex flax, and (c) BioMid fibre	sandwich core	2016	[30]
N/A	core: kenaf, hemp shell: jute/flax (Biotex)	preform Shell	2017	[78]
*Alaska white rot*	Alaska birch (Betula neoalaskana), millet grain, wheat bran, natural fibre, calcium sulfate	backfill/structure	2017	[23]
*Basidiomycetes*	agricultural byproducts: cotton (leaves, sticks, burs); switchgrass, rice straw, sorghum stalks, cotton burs, kenaf and corn stalks	acoustic insulation	2017	[79]
*G. lucidum, P. ostreatus*	cellulose, cellulose & potato-dextrose (PDB)	mycelium films	2017	[13]
N/A	sawdust or agricultural waste, nutrients (not specified)	furniture	2017	[54]
*T. versicolor*	rice hulls, wheat grain inoculum	insulating foams	2017	[10]
*P. pulmonarius, P. ostreatus, P. salmoneo, A. agrocibe*	agricultural byproducts: woodchips of eucalyptus, oak, pine and apple	composite & biopolymer	2017	[27]
N/A	skin: natural fibre textile (jute, hemp and cellulose). core: pre-grown kenaf–hemp mixtures	laminated bio-composite	2017	[55]
*Basidiomycetes*	agricultural waste: Corn stover particles; Calcium and carbohydrate (not specified)	composite & biopolymer	2017	[80]
(*Ecovative*)	calcium and carbohydrate (not specified)	synthetic polymer alternatives	2017	[12]
*P. ostreatus*	seeds (not specified) mixed with hydrogel	architectural assembly units	2017	[81]
*S. commune*	broth culture, agar minimal medium	thermoplastic alternative	2018	[17]
*T. versicolor*	rice hulls, glass fines, wheat grains	insulation, furniture, building	2018	[82]
*O. latermarginatus, M. minor, G. resinaceum*	wheat straw	insulation materials	2018	[83]
*P. ostreatus, T. multicolor*	rapeseed straw, beech sawdust, non-woven cotton fibres	product design	2018	[42]
N/A	N/A	laminated bio-composite	2018	[84]
*Trametes* sp. *S. Commune*	bread particles, banana peel, coffee residue, Styrofoam pellets, flower, orange peel, carrot leaves, cardboard, sawdust, straw	product design	2018	[35]
(*Ecovative*)	calcium and carbohydrate (not specified)	N/A	2018	[85]
*T. versicolor*	hemp, flax, flax waste, softwood, straw, varied processing: loose, chopped, dust, pre-compressed and tow	building materials	2019	[20]
(*Ecovative*)	a mixture of spruce, pine, and fir (SPF) particleboard particles	packaging and furniture	2019	[86]
*L. edodes isolates*	coconut powder-based supplemented with wheat bran	packaging	2019	[87]
*F. pinicola, G. sepiarium,* *L. sulphureus, P. schweinitzii, P. betulinus, P. ostreatus, P. arcularius,* *T. pubescens, T. suaveolens, T. abietinum*	wood shavings of *Betula papyrifera* (Birch), *Populus tremuloides* (Aspen), *Picea glauca* (Spruce), *Pinus contorta* (Pine), *Abies lasiocarpa* (Fir). Addition of nutrient solution: peptone, malt extract, and yeast	thermal insulation boards	2019	[88]
*C.versicolor T. multicolor G. sesille*	vine and apple tree-pruning woodchips mixed with mixed with 1% flour and 3% wheat straw	thermal insulation water container	2019	[24]
*P. sanguineus, P. albidus, L. velutinus*	wood sawdust, wheat bran and calcium carbonate	EPS alternative	2019	[89]
*Lentinula edodes*	peach-palm residues, ammonium sulphate, potassium nitrate, and cooked soy flour	evaluation of MBC physico-chemical, enzymatic activities, thermal and mechanical properties	2020	[90]
*Pleurotus ostreatus*	wheat bran, sugarcane, sawdust	bio-blocks, sustainable applications	2020	[91]
*Ganoderma lucidum*	bamboo fibre	development of an extrudable and buildable composite	2020	[92]
*Ganoderma lucidum*	cotton stalk	property improvement of MBC	2020	[93]
*Aurantiporus, Ganoderma, Lentinus, Pleurotus ostreatus and Panus* sp.	cultivated on PDA, PDB	biotechnological applications	2021	[94]
*Trametes versicolor, Ganoderma resinaceum*	hemp hurds, beechwood sawdust	formwork application	2021	[95]
*Wood decay basidiomycete*	hemp shive, cotton	enhancement of MBC	2021	[96]
*Pleurotus Ostreatus*	saw dust-coir pith	packaging	2021	[97]
N/A	silica compounds	glass microchannels fabrication	2021	[98]
*P. ostreatus*	rice husk	Bio-foam	2021	[99]
*Pleurotus ostreatus*	polyacrylonitrile (PAN) nano-fibre mats	reinforced nanocomposites	2021	[100]
*Abortiporus biennis, Bjerkandera adusta, Coriolopsis gallica, Coriolopsis gallica, Coriolopsis trogii, Daedaleopsis confragosa, Daedaleopsis tricolor, Fomes fomentarius, Fomitiporia mediterranea, Fomitopsis iberica, Fomitopsis pinicola, Ganoderma carnosum, Ganoderma lucidum, Irpex lacteus, Irpiciporus pachyodon, Lenzites betulinus, Neofavolus alveolaris, Stereum hirsutum, erana caerulea, Trametes hirsuta, Trametes suaveolens*	millet grains	MBC development	2021	[101]
*Ganoderma resinaceum*	waste Rose flower and Lavender straw	MBC development	2021	[102]
*Pleurotus ostreatus*	sawdust, bagasse, and coffee husk	construction	2022	[103]
N/A	strawbale, wood shavings, coffee grounds	muti-organism composite	2022	[104]
*Aspergillus flavus*	N/A	visual lateral flow immunoassays/bioanalysis	2022	[105]
*Ganoderma lucidum*	hemp fibres, hemp hurds, pine wood sawdust and shavings, and silvergrass (*Miscanthus*) shavings	building materials	2022	[106]
*Pleurotus ostreatus*	waste cardboard, paper, and newsprint substrates	sound absorption properties study	2022	[107]
*Ganoderma lucidum*	beechwood sawdust	robotic manufacturing	2022	[108]
*Pleurotus ostreatus*	wood plugs, hemp fibres, wood chips	fibre-reinforced composite fabrication	2022	[109]
*Lentinus crinitus*	barley straw	fabrication of insulation panels	2022	[110]
*Ganoderma fornicatum, Ganoderma williamsianum, Lentinus sajor-caju, Schizophyllum commune*	sawdust, corn husk, and rice straw	chemical, physical and mechanical properties investigation	2022	[111]
*Trametes versicolor*	hemp fibres	evaluation of nano-clay effect on MBC properties	2022	[112]
*Aspergillus terreus*	silver salt solution, PDA, PDB	silver nanoparticles fabrication	2022	[113]
*Streptomyces*	calcium alginate, YGM medium	polymeric encapsulation	2023	[114]
*Trametes versicolor, Pleurotus ostreatus, P. eryngii, Ganoderma carnosum and Fomitopsis pinicola*	millet, wheat and a 1:1 mix of millet and wheat grains	insulation panels	2023	[115]
*Pleurotus ostreatus*	malt extract agar & activated charcoal	single-layer masks	2023	[116]
N/A	N/A	generating Gradient porous structures (GPS)	2023	[117]
*Pleurotus ostreatus*	spent coffee grounds, natural pineapple fibres (NPFs)	MBC fabrication	2023	[118]
*Ganoderma lucidum (Reishi), Oyster mushrooms*	N/A	tool design	2023	[119]
N/A	N/A	environmental evaluation	2024	[120]
*Ganoderma fornicatum, Ganoderma williamsianum, Lentinus sajor-caju, Trametes coccinea*	bamboo sawdust & corn pericarp	modern interior material	2024	[121]
*Lentinus sajor-caju*	corn husk and sawdust	MBC development	2024	[122]
*Aspergillus niger*	coating agents: Au nanoparticles, borohydride, glucose, citrate, and an antibiotic	biosensing and environmental applications	2024	[123]
*Ganoderma lucidum*	sawdust	MBC fabrication	2024	[124]
*Lentinus sajor-caju*	various ratios of corn husk and sawdust	MBC development	2024	[125]

N/A refers to no available data.

**Table 2:** A comparison of MBC physical properties (non-compressed) with other conventional materials.

Material	Density (kg/m^3^)	Water Absorption (%)	Dimensional Stability (%)	Thermal Resistance (K·m^2^/W)
Mycelium-based composites	59–318 [10]	300 [141]	0.64–2.4 [141]	0.82–1.5 [141]
BC-mycelium composite [151]	1208–2857	-	-	-
Plywood	512–596 [152]	5–49 [26]	-	0.084–0.1 [152]
Standard EPS board [153]	12–48	0.3–4	<2	0.55–0.88
Polystyrene foams	22–30 [154]	0.03–9 [26]	-	0.32–0.35 [155]

**Table 5:** Basic cost of constructing a house with different building materials [209].

Variables	Base Values (USD)
Price of mycelium/ft3	$0.83
Plexiglass	$190.53
Strip plexiglass	$25.81
Plywood	$40.00
Strip plywood	$1.25
Interest rate	7.75%
CPI (inflation rate)	6.50%
Concrete house	$61,873.00
Lumber house	$61,200.00
Mycelium-Plywood house	$17,263.75
Mycelium-Plexiglass house	$59,810.62

**Table 6:** The eco-costs and life cycle assessment of construction materials over the duration of their lifespan [4,154,223,224].

Material	Lifespan (Year)	Eco-Costs (Euro)	Eco-Costs in 500 Years (Euro)	Fossil Energy Demand (MJ)	Climate Change (kg CO_2_)
Concrete	80–150	792	2640	7.47	0.5425
Mycelium	<50	16	160	7.26	0.6417
CoRncrete	50	168	1680	-	-
Hempcrete	>500	78	78	7.71	0.6933
Bio-Bricks	200	245	612.5	-	-

## Abbreviations

The following abbreviations are used in this manuscript:

MBC	Mycelium-based Composite
MDD	Material Driven Design
PDA	Potato Dextrose Agar
PDB	Potato Dextrose Broth
H_2_O_2_	Hydrogen Peroxide
BC	Bacterial Cellulose
MBF	Mycelium-based Foam
NRPs	Natural Reinforcing Particles
PFA	Polyfurfuryl Alcohol
SiO_2_	Silica
PMMA	Poly Methyl Methacrylate
PLA FTIR	Polylactic Acid Fourier Transform Infrared
TGA	Thermogravimetric Analysis
SEM	Scanning Electron Microscopy
CPI	Consumer Price Index
LCA	Life Cycle Assessment
GWP	Global Warming Potential
GHG	Greenhouse Gas

## References

[1] Madurwar M.V., Ralegaonkar R.V., Mandavgane S.A. (2013). Application of agro-waste for sustainable construction materials: A review. Constr. Build. Mater..

[2] Pheng S., Premnath R. (2019). Construction Quality and the Economy.

[3] Sahu M.K., Singh L., Choudhary S.N. (2016). Critical review on bricks. Int. J. Eng. Manag. Res..

[4] Sharma R., Sumbria R. (2022). Mycelium bricks and composites for sustainable construction industry: A state-of-the-art review. Innov. Infrastruct. Solut..

[5] Bhuvaneshwari S., Hettiarachchi H., Meegoda J.N. (2019). Crop residue burning in India: Policy challenges and potential solutions. Int. J. Environ. Res. Public Health.

[6] Defonseka C. (2019). Polymeric Composites with Rice Hulls: An Introduction.

[7] Javadian A., Le Ferrand H., Hebel D.E., Saeidi N. (2020). Application of mycelium-bound composite materials in construction industry: A short review. SOJ Mater. Sci. Eng..

[8] Lingam D., Narayan S., Mamun K., Charan D. (2023). Engineered mycelium-based composite materials: Comprehensive study of various properties and applications. Constr. Build. Mater..

[9] Kalka S., Huber T., Steinberg J., Baronian K., Müssig J., Staiger M.P. (2014). Biodegradability of all-cellulose composite laminates. Compos. Part A Appl. Sci. Manuf..

[10] Jones M., Huynh T., Dekiwadia C., Daver F., John S. (2017). Mycelium composites: A review of engineering characteristics and growth kinetics. J. Bionanosci..

[11] Nawawi W.M., Jones M., Murphy R.J., Lee K.-Y., Kontturi E., Bismarck A. (2019). Nanomaterials derived from fungal sources—Is it the new hype?. Biomacromolecules.

[12] Islam M.R., Tudryn G., Bucinell R., Schadler L., Picu R. (2017). Morphology and mechanics of fungal mycelium. Sci. Rep..

[13] Haneef M., Ceseracciu L., Canale C., Bayer I.S., Guerrero J.A.H., Athanassiou A. (2017). Advanced materials from fungal mycelium: Fabrication and tuning of physical properties. Sci. Rep..

[14] Swift R.S. (1996). Organic matter characterization. Methods of Soil Analysis: Part 3 Chemical Methods.

[15] Sydor M., Cofta G., Doczekalska B., Bonenberg A. (2022). Fungi in mycelium-based composites: Usage; recommendations. Materials.

[16] Victoria S. Market Summary–Recycled Glass. Retrieved May 2014. https://assets.sustainability.vic.gov.au/susvic/Report-Market-Analysis-Glass-Sept-2014.pdf.

[17] Appels F.V., Camere S., Montalti M., Karana E., Jansen K.M., Dijksterhuis J., Wösten H.A. (2019). Fabrication factors influencing mechanical, moisture-and water-related properties of mycelium-based composites. Mater. Des..

[18] Zimele Z., Irbe I., Grinins J., Bikovens O., Verovkins A., Bajare D. (2020). Novel mycelium-based biocomposites (Mbb) as building materials. J. Renew. Mater..

[19] Deepa B., Abraham E., Cordeiro N., Mozetic M., Mathew A.P., Oksman K., Pothan L.A. (2015). Utilization of various lignocellulosic biomass for the production of nanocellulose: A comparative study. Cellulose.

[20] Elsacker E., Vandelook S., Brancart J., Peeters E., De Laet L. (2019). Mechanical, physical and chemical characterisation of mycelium-based composites with different types of lignocellulosic substrates. PLoS ONE.

[21] Abhijith R., Ashok A., Rejeesh C. (2018). Sustainable packaging applications from mycelium to substitute polystyrene: A review. Mater. Today Proc..

[22] Shanmugam V., Mensah R.A., Försth M., Sas G., Restás Á., Addy C., Singha S. (2021). Circular economy in biocomposite development: State-of-the-art, challenges and emerging trends. Compos. Part C Open Access.

[23] Yang Z., Zhang F., Still B., White M., Amstislavski P. (2017). Physical and mechanical properties of fungal mycelium-based biofoam. J. Mater. Civ. Eng..

[24] Attias N., Danai O., Tarazi E., Pereman I., Grobman Y.J. (2019). Implementing bio-design tools to develop mycelium-based products. Des. J..

[25] Shakir M.A., Azahari B., Yusup Y., Yhaya M.F., Salehabadi A., Ahmad M.I. (2020). Preparation and characterization of mycelium as a bio-matrix in fabrication of bio-composite. J. Adv. Res. Fluid Mech. Therm. Sci..

[26] Jones M., Mautner A., Luenco S., Bismarck A., John S. (2020). Engineered mycelium composite construction materials from fungal biorefineries: A critical review. Mater. Des..

[27] Attias N., Danai O., Ezov N., Tarazi E., Grobman Y.J. Developing novel applications of mycelium based bio-composite materials for design and architecture. Proceedings of the Building with Biobased Materials: Best Practice and Performance Specification.

[28] Gezer E.D., Gümüşkaya E., Uçar E., Ustaömer D. (2020). Mechanical properties of mycelium based MDF. Sigma J. Eng. Nat. Sci..

[29] Elsacker E., Vandelook S., Van Wylick A., Ruytinx J., De Laet L., Peeters E. (2020). A comprehensive framework for the production of mycelium-based lignocellulosic composites. Sci. Total Environ..

[30] Jiang L., Walczyk D., McIntyre G., Bucinell R. (2016). A new approach to manufacturing biocomposite sandwich structures: Mycelium-based cores. International Manufacturing Science and Engineering Conference.

[31] Zhao S., Shen Y., Jiang X., Lv H., Han C., Zhao Q. (2024). A critical review of clay mineral-based photocatalysts for wastewater treatment. Catalysts.

[32] Gao D.-c., Sun Y., Fong A.M., Gu X. (2022). Mineral-based form-stable phase change materials for thermal energy storage: A state-of-the art review. Energy Storage Mater..

[33] Syduzzaman M., Al Faruque M.A., Bilisik K., Naebe M. (2020). Plant-based natural fibre reinforced composites: A review on fabrication, properties and applications. Coatings.

[34] Courard L., Vallas T. (2017). Using nature in architecture: Building a living house with mycelium and living trees. Front. Archit. Res..

[35] Karana E., Blauwhoff D., Hultink E.-J., Camere S. (2018). When the material grows: A case study on designing (with) mycelium-based materials. Int. J. Des..

[36] Santhosh B., Bhavana D., Rakesh M. (2018). Mycelium composites: An emerging green building material. Int. Res. J. Eng. Technol..

[37] Ghazvinian A., Farrokhsiar P., Vieira F., Pecchia J., Gursoy B. (2019). Mycelium-based bio-composites for architecture: Assessing the effects of cultivation factors on compressive strength. Mater. Res. Inno..

[38] Blauwhoff D. (2016). Mycelium Based Materials: A Study on Material Driven Design and Forecasting Acceptance. Master’s Thesis.

[39] Rinaudo M. (2006). Chitin and chitosan: Properties and applications. Prog. Polym. Sci..

[40] Bartnicki-Garcia S. (1968). Cell wall chemistry; morphogenesis, and taxonomy of fungi. Annu. Rev. Microbiol..

[41] Wessels J., Mol P., Sietsma J., Vermeulen C. (1990). Wall structure, wall growth, and fungal cell morphogenesis. Biochemistry of Cell Walls and Membranes in Fungi.

[42] Appels F.V., Dijksterhuis J., Lukasiewicz C.E., Jansen K.M., Wösten H.A., Krijgsheld P. (2018). Hydrophobin gene deletion and environmental growth conditions impact mechanical properties of mycelium by affecting the density of the material. Sci. Rep..

[43] Zabel R., Morrell J. (2020). The characteristics and classification of fungi and bacteria. Wood Microbiology.

[44] Geoghegan I., Steinberg G., Gurr S. (2017). The role of the fungal cell wall in the infection of plants. Trends Microbiol..

[45] Prasher I. (2015). Wood-Rotting Non-Gilled Agaricomycetes of Himalayas.

[46] Pegler D. (1996). Hyphal analysis of basidiomata. Mycol. Res..

[47] Bayer E., McIntyre G. (2012). Substrate Composition and Method for Growing Mycological Materials. U.S. Patent.

[48] Islam M.R., Omar M., Moyen Uddin M.P.K., Phytochemicals K. (2015). *Ganoderma lucidum* and *Lentinula edodes* accessible in Bangladesh. Am. J. Biol. Life Sci..

[49] Pk U., Talukder R.I., Sarkar M.K., Rahman T., Pervin R., Rahman M., Akther L. (2019). Effect of Solvents on Phytochemicals Content and Antioxidant Activity of *Ganoderma lucidum*. Open Microbiol. J..

[50] Petre M. (2015). Mushroom Biotechnology: Developments and Applications.

[51] Bayer E., McIntyre G.R. (2016). Method for Growing Mycological Materials. U.S. Patent.

[52] Parisi S., Rognoli V., Garcia C.A. (2016). Designing materials experiences through passing of time: Material driven design method applied to mycelium-based composites. Celebration & Contemplation: Proceedings of the 10th International Conference on Design and Emotion 2016.

[53] Ziegler A.R., Bajwa S.G., Holt G.A., McIntyre G., Bajwa D.S. (2016). Evaluation of physico-mechanical properties of mycelium reinforced green biocomposites made from cellulosic fibers. Appl. Eng. Agric..

[54] Dahmen J. (2017). Soft futures: Mushrooms and regenerative design. J. Archit. Educ..

[55] Jiang L., Walczyk D., McIntyre G., Bucinell R., Tudryn G. (2017). Manufacturing of biocomposite sandwich structures using mycelium-bound cores and preforms. J. Manuf. Process..

[56] Girometta C., Picco A.M., Baiguera R.M., Dondi D., Babbini S., Cartabia M., Savino E. (2019). Physico-mechanical and thermodynamic properties of mycelium-based biocomposites: A review. Sustainability.

[57] Golak-Siwulska I., Kałużewicz A., Spiżewski T., Siwulski M., Sobieralski K. (2018). Bioactive compounds and medicinal properties of Oyster mushrooms (*Pleurotus* sp.). Folia Hortic..

[58] Josephine R. (2015). A review on oyster mushroom (*Pleurotus* spp.). Int. J. Curr. Res..

[59] Sharma M., Verma S., Chauhan G., Arya M., Kumari A. (2014). Mycelium-based biocomposites: Synthesis and applications. Environ. Sustain..

[60] Aiduang W., Chanthaluck A., Kumla J., Jatuwong K., Srinuanpan S., Waroonkun T., Suwannarach N. (2022). Amazing fungi for eco-friendly composite materials: A comprehensive review. J. Fungi.

[61] Suwannarach N., Kumla J., Zhao Y., Kakumyan P. (2022). Impact of cultivation substrate and microbial community on improving mushroom productivity: A review. Biology.

[62] Rana S., Rana R., Thapliyal D., Verma S., Mehra A., Bhargava C.K., Arya R.K. (2024). Potential Exploitation of Agro-Industrial Waste. From Waste to Wealth.

[63] Wan B., Dong F., Chen M., Zhu J., Tan J., Fu X., Chen S. (2016). Advances in recycling and utilization of agricultural wastes in China: Based on environmental risk, crucial pathways, influencing factors, policy mechanism. Procedia Environ. Sci..

[64] Xue L., Zhang P., Shu H., Wang R., Zhang S. (2016). Agricultural waste. Water Environ. Res..

[65] Chattopadhyay S., Dutta A., Ray S. (2009). Municipal solid waste management in Kolkata, India—A review. Waste Manag..

[66] Hoornweg D., Bhada-Tata P. (2012). What A Waste: A Global Review of Solid Waste Management.

[67] Sadh P.K., Duhan S., Duhan J.S. (2018). Agro-industrial wastes and their utilization using solid state fermentation: A review. Bioresour. Bioprocess..

[68] Antinori M.E., Contardi M., Suarato G., Armirotti A., Bertorelli R., Mancini G., Athanassiou A. (2021). Advanced mycelium materials as potential self-growing biomedical scaffolds. Sci. Rep..

[69] Paul V., Kanny K., Redhi G.G. (2015). Mechanical, thermal and morphological properties of a bio-based composite derived from banana plant source. Compos. Part A Appl. Sci. Manuf..

[70] Jiang L., Walczyk D., McIntyre G. A new process for manufacturing biocomposite laminate and sandwich parts using mycelium as a binder. Proceedings of the Applied Superconductivity Conference (ASC 2014).

[71] Jiang L., Walczyk D.F., McIntyre G. Vacuum infusion of mycelium-bound biocomposite preforms with natural resins. Proceedings of the CAMX Conference Proceedings.

[72] Cheng C.M., Su D.G., Zhong M.F. (2014). Study on the mechanical properties of the latex-mycelium composite. Appl. Mech. Mater..

[73] Travaglini S., Dharan C., Ross P. (2014). Mycology matrix sandwich composites flexural characterization. Proceedings of American Society for Composites.

[74] Travaglini S., Dharan C., Ross P.G. Thermal properties of mycology materials. Proceedings of the American Society of Composites-30th Technical Conference.

[75] Lelivelt R., Lindner G., Teuffel P., Lamers H. The production process and compressive strength of mycelium-based materials. Proceedings of the First International Conference on Bio-based Building Materials.

[76] Travaglini S., Dharan C., Ross P. Manufacturing of mycology composites. Proceedings of the American Society for Composites: Thirty-First Technical Conference.

[77] Mayoral González E., Gonzalez Diez I. (2016). Bacterial induced cementation processes and mycelium panel growth from agricultural waste. Key Eng. Mater..

[78] Jiang L., Walczyk D., McIntyre G. (2017). A new approach to manufacturing biocomposite sandwich structures: Investigation of preform shell behavior. J. Manuf. Sci. Eng..

[79] Pelletier M., Holt G., Wanjura J., Lara A., Tapia-Carillo A., McIntyre G., Bayer E. (2017). An evaluation study of pressure-compressed acoustic absorbers grown on agricultural by-products. Ind. Crops Prod..

[80] Tudryn G.J., Smith L.C., Freitag J., Bucinell R., Schadler L.S. (2018). Processing and morphology impacts on mechanical properties of fungal based biopolymer composites. J. Polym. Environ..

[81] Campbell S., Correa D., Wood D., Menges A. Modular Mycelia: Scaling Fungal Growth for Architectural Assembly. Proceedings of the Computational Fabrication—eCAADe RIS.

[82] Jones M., Bhat T., Huynh T., Kandare E., Yuen R., Wang C.H., John S. (2018). Waste-derived low-cost mycelium composite construction materials with improved fire safety. Fire Mater..

[83] Xing Y., Brewer M., El-Gharabawy H., Griffith G., Jones P. (2018). Growing and testing mycelium bricks as building insulation materials. IOP Conference Series: Earth and Environmental Science: Earth And Environmental Science.

[84] Jiang L., Walczyk D., McIntyre G., Bucinell R., Li B. (2019). Bioresin infused then cured mycelium-based sandwich-structure biocomposites: Resin transfer molding (RTM) process, flexural properties, and simulation. J. Clean. Prod..

[85] Islam M., Tudryn G., Bucinell R., Schadler L., Picu R. (2018). Stochastic continuum model for mycelium-based bio-foam. Mater. Des..

[86] Sun W., Tajvidi M., Hunt C.G., McIntyre G., Gardner D.J. (2019). Fully bio-based hybrid composites made of wood, fungal mycelium and cellulose nanofibrils. Sci. Rep..

[87] Matos M.P., Teixeira J.L., Nascimento B.L., Griza S., Holanda F.S.R., Marino R.H. (2019). Production of biocomposites from the reuse of coconut powder colonized by Shiitake mushroom. Ciência Agrotecnolog..

[88] Wimmers G., Klick J., Tackaberry L., Zwiesigk C., Egger K., Massicotte H. (2019). Fundamental studies for designing insulation panels from wood shavings and filamentous fungi. BioResources.

[89] Bruscato C., Malvessi E., Brandalise R.N., Camassola M. (2019). High performance of macrofungi in the production of mycelium-based biofoams using sawdust—Sustainable technology for waste reduction. J. Clean. Prod..

[90] de Lima G.G., Schoenherr Z.C.P., Magalhães W.L.E., Tavares L.B.B., Helm C.V. (2020). Enzymatic activities and analysis of a mycelium-based composite formation using peach palm (*Bactris gasipaes*) residues on Lentinula edodes. Bioresour. Bioprocess..

[91] Joshi K., Meher M.K., Poluri K.M. (2020). Fabrication and characterization of bioblocks from agricultural waste using fungal mycelium for renewable and sustainable applications. ACS Appl. Bio Mater..

[92] Soh E., Chew Z.Y., Saeidi N., Javadian A., Hebel D., Le Ferrand H. (2020). Development of an extrudable paste to build mycelium-bound composites. Mater. Des..

[93] Liu R., Li X., Long L., Sheng Y., Xu J., Wang Y. (2020). Improvement of mechanical properties of mycelium/cotton stalk composites by water immersion. Compos. Interfaces.

[94] César E., Canche-Escamilla G., Montoya L., Ramos A., Duarte-Aranda S., Bandala V.M. (2021). Characterization and physical properties of mycelium films obtained from wild fungi: Natural materials for potential biotechnological applications. J. Polym. Environ..

[95] Elsacker E., Søndergaard A., Van Wylick A., Peeters E., De Laet L. (2021). Growing living and multifunctional mycelium composites for large-scale formwork applications using robotic abrasive wire-cutting. Constr. Build. Mater..

[96] Sisti L., Gioia C., Totaro G., Verstichel S., Cartabia M., Camere S., Celli A. (2021). Valorization of wheat bran agro-industrial byproduct as an upgrading filler for mycelium-based composite materials. Ind. Crops Prod..

[97] Sivaprasad S., Byju S.K., Prajith C., Shaju J., Rejeesh C. (2021). Development of a novel mycelium bio-composite material to substitute for polystyrene in packaging applications. Mater. Today Proc..

[98] Sato D., Tsumori F. (2021). Glass Microchannel Formation by Mycelium. J. Photopolym. Sci. Technol..

[99] Nashiruddin N.I., Chua K.S., Mansor A.F., Rahman R.A., Lai J.C., Wan Azelee N.I., El Enshasy H. (2022). Effect of growth factors on the production of mycelium-based biofoam. Clean Technol. Environ. Policy.

[100] Trabelsi M., Mamun A., Klöcker M., Brockhagen B., Kinzel F., Kapanadze D., Sabantina L. (2021). Polyacrylonitrile (PAN) nanofiber mats for mushroom mycelium growth investigations and formation of mycelium-reinforced nanocomposites. J. Eng. Fibers Fabr..

[101] Cartabia M., Girometta C.E., Milanese C., Baiguera R.M., Buratti S., Branciforti D.S., Vadivel D., Girella A., Babbini S., Savino E. (2021). Collection and characterization of wood decay fungal strains for developing pure mycelium mats. J. Fungi.

[102] Angelova G., Brazkova M., Stefanova P., Blazheva D., Vladev V., Petkova N., Slavov A., Denev P., Karashanova D., Zaharieva R. (2021). Waste rose flower and lavender straw biomass—An innovative lignocellulose feedstock for mycelium bio-materials development using newly isolated Ganoderma resinaceum GA1M. J. Fungi.

[103] Alemu D., Tafesse M., Gudetta Deressa Y. (2022). Production of mycoblock from the mycelium of the fungus Pleurotus ostreatus for use as sustainable construction materials. Adv. Mater. Sci. Eng..

[104] Hoenerloh A., Ozkan D., Scott J. (2022). Multi-organism composites: Combined growth potential of mycelium and bacterial cellulose. Biomimetics.

[105] Liang M., Cai X., Gao Y., Yan H., Fu J., Tang X., Zhang Q., Li P. (2022). A versatile nanozyme integrated colorimetric and photothermal lateral flow immunoassay for highly sensitive and reliable Aspergillus flavus detection. Biosens. Bioelectron..

[106] Özdemir E., Saeidi N., Javadian A., Rossi A., Nolte N., Ren S., Dwan A., Acosta I., Hebel D.E., Wurm J. (2022). Wood-veneer-reinforced mycelium composites for sustainable building components. Biomimetics.

[107] Walter N., Gürsoy B. (2022). A study on the sound absorption properties of mycelium-based composites cultivated on waste paper-based substrates. Biomimetics.

[108] Elsacker E., Peeters E., De Laet L. (2022). Large-scale robotic extrusion-based additive manufacturing with living mycelium materials. Sustain. Futures.

[109] Nguyen M.T., Solueva D., Spyridonos E., Dahy H. (2022). Mycomerge: Fabrication of mycelium-based natural fiber reinforced composites on a rattan framework. Biomimetics.

[110] César E., Montoya L., Bárcenas-Pazos G.M., Ordóñez-Candelaria V.R., Bandala V.M. (2021). Performance of mycelium composites of Lentinus crinitus under two compression protocols. Madera Bosques.

[111] Aiduang W., Kumla J., Srinuanpan S., Thamjaree W., Lumyong S., Suwannarach N. (2022). Mechanical, physical, and chemical properties of mycelium-based composites produced from various lignocellulosic residues and fungal species. J. Fungi.

[112] Elsacker E., De Laet L., Peeters E. (2022). Functional grading of mycelium materials with inorganic particles: The effect of nanoclay on the biological, chemical and mechanical properties. Biomimetics.

[113] Yari T., Vaghari H., Adibpour M., Jafarizadeh-Malmiri H., Berenjian A. (2022). Potential application of Aspergillus terreus, as a biofactory, in extracellular fabrication of silver nanoparticles. Fuel.

[114] Mancera-López M.E., Barrera-Cortés J., Mendoza-Serna R., Ariza-Castolo A., Santillan R. (2022). Polymeric encapsulate of Streptomyces mycelium resistant to dehydration with air flow at room temperature. Polymers.

[115] Charpentier-Alfaro C., Benavides-Hernández J., Poggerini M., Crisci A., Mele G., Della Rocca G., Emiliani G., Frascella A., Torrigiani T., Palanti S. (2023). Wood-decaying fungi: From timber degradation to sustainable insulating biomaterials production. Materials.

[116] French V., Du C., Foster E.J. (2023). Mycelium as a self-growing biobased material for the fabrication of single-layer masks. J. Bioresour. Bioprod..

[117] Olivero E., Gawronska E., Manimuda P., Jivani D., Chaggan F.Z., Corey Z., de Almeida T.S., Kaplan-Bie J., McIntyre G., Wodo O. (2023). Gradient porous structures of mycelium: A quantitative structure–mechanical property analysis. Sci. Rep..

[118] Kohphaisansombat C., Jongpipitaporn Y., Laoratanakul P., Tantipaibulvut S., Euanorasetr J., Rungjindamai N., Chuaseeharonnachai C., Kwantong P., Somrithipol S., Boonyuen N. (2023). Fabrication of mycelium (oyster mushroom)-based composites derived from spent coffee grounds with pineapple fibre reinforcement. Mycology.

[119] Gough P., Perera P.B., Kertesz M.A., Withana A. Design, Mould, Grow!: A Fabrication Pipeline for Growing 3D Designs Using Myco-Materials. Proceedings of the 2023 CHI Conference on Human Factors in Computing Systems.

[120] Bagheriehnajjar G., Yousefpour H., Rahimnejad M. (2024). Environmental impacts of mycelium-based bio-composite construction materials. Int. J. Environ. Sci. Technol..

[121] Aiduang W., Jatuwong K., Jinanukul P., Suwannarach N., Kumla J., Thamjaree W., Lumyong S. (2024). Sustainable Innovation: Fabrication and characterization of mycelium-based green composites for modern interior materials using agro-industrial wastes and different species of fungi. Polymers.

[122] Teeraphantuvat T., Jatuwong K., Jinanukul P., Thamjaree W., Lumyong S., Aiduang W. (2024). Improving the physical and mechanical properties of mycelium-based green composites using paper waste. Polymers.

[123] Sadaf A., Afolayan J.S., Perry C.C. (2024). Developing gold nanoparticle mycelial composites: Effect of nanoparticle surface functionality on Aspergillus niger viability and cell wall biochemistry. Curr. Res. Biotechnol..

[124] Wang H., Tao J., Wu Z., Weiland K., Wang Z., Masania K., Wang B. (2024). Fabrication of Living Entangled Network Composites Enabled by Mycelium. Adv. Sci..

[125] Jinanukul P., Kumla J., Aiduang W., Thamjaree W., Oranratmanee R., Shummadtayar U., Tongtuam Y., Lumyong S., Suwannarach N., Waroonkun T. (2024). Comparative Evaluation of Mechanical and Physical Properties of Mycelium Composite Boards Made from *Lentinus sajor-caju* with Various Ratios of Corn Husk and Sawdust. J. Fungi.

[126] Butu A., Rodino S., Miu B., Butu M. (2020). Mycelium-based materials for the ecodesign of bioeconomy. Dig. J. Nanomater. Biostruct.

[127] Kumla J., Suwannarach N., Sujarit K., Penkhrue W., Kakumyan P., Jatuwong K., Vadthanarat S., Lumyong S. (2020). Cultivation of mushrooms and their lignocellulolytic enzyme production through the utilization of agro-industrial waste. Molecules.

[128] Bellettini M.B., Fiorda F.A., Maieves H.A., Teixeira G.L., Ávila S., Hornung P.S., Júnior A.M., Ribani R.H. (2019). Factors affecting mushroom *Pleurotus* spp.. Saudi J. Biol. Sci..

[129] Kuribayashi T., Lankinen P., Hietala S., Mikkonen K.S. (2022). Dense and continuous networks of aerial hyphae improve flexibility and shape retention of mycelium composite in the wet state. Compos. Part A Appl. Sci. Manuf..

[130] Agustina W., Aditiawati P., Kusumah S., Dungani R. (2019). Physical and mechanical properties of composite boards from the mixture of palm sugar fiber and cassava bagasse using mycelium of *Ganoderma lucidum* as a biological adhesive. Proceedings of IOP Conference Series: Earth and Environmental Science.

[131] Hoa H.T., Wang C.-L. (2015). The effects of temperature and nutritional conditions on mycelium growth of two oyster mushrooms (*Pleurotus ostreatus* and *Pleurotus cystidiosus*). Mycobiology.

[132] Velasco P.M., Ortiz M.P.M., Giro M.A.M., Castelló M.C.J., Velasco L.M. (2014). Development of better insulation bricks by adding mushroom compost wastes. Energy Build..

[133] Deacon J.W. (2005). Fungal Biology.

[134] Attias N., Danai O., Abitbol T., Tarazi E., Ezov N., Pereman I., Grobman Y.J. (2020). Mycelium bio-composites in industrial design and architecture: Comparative review and experimental analysis. J. Clean. Prod..

[135] Silverman J., Cao H., Cobb K. (2020). Development of mushroom mycelium composites for footwear products. Cloth. Text. Res. J..

[136] Cerimi K., Akkaya K.C., Pohl C., Schmidt B., Neubauer P. (2019). Fungi as source for new bio-based materials: A patent review. Fungal Biol. Biotechnol..

[137] Gortner F., Schüffler A., Fischer-Schuch J., Mitschang P. (2022). Use of bio-based and renewable materials for sheet molding compounds (SMC)—Mechanical properties and susceptibility to fungal decay. Compos. Part C Open Access.

[138] Taylor E.C. (1979). Seasonal distribution and abundance of fungi in two desert grassland communities. J. Arid. Environ..

[139] Rowan N.J., Johnstone C.M., McLean R.C., Anderson J.G., Clarke J.A. (1999). Prediction of toxigenic fungal growth in buildings by using a novel modelling system. Appl. Environ. Microbiol..

[140] Lelivelt R. (2015). The mechanical possibilities of mycelium materials. Eindh. Univ. Technol. (TU/E).

[141] Holt G.A., Mcintyre G., Flagg D., Bayer E., Wanjura J., Pelletier M. (2012). Fungal mycelium and cotton plant materials in the manufacture of biodegradable molded packaging material: Evaluation study of select blends of cotton byproducts. J. Biobased Mater. Bioenergy.

[142] Bjornhov T., Ljungh J., Olsson R. (2010). Method for Producing a Composite Material. U.S. Patent.

[143] Griffin D.H. (1996). Fungal Physiology.

[144] Jones M.P., Lawrie A.C., Huynh T.T., Morrison P.D., Mautner A., Bismarck A., John S. (2019). Agricultural by-product suitability for the production of chitinous composites and nanofibers utilising *Trametes versicolor* and *Polyporus brumalis* mycelial growth. Process Biochem..

[145] Gibson I., Ashby M.F. (1982). The mechanics of three-dimensional cellular materials. Proc. R. Soc. London A Math. Phys. Sci..

[146] Dai C., Yu C., Zhou X. (2005). Heat and mass transfer in wood composite panels during hot pressing. Part II. Modeling void formation and mat permeability. Wood Fiber Sci..

[147] Qin Z., Jung G.S., Kang M.J., Buehler M.J. (2017). The mechanics and design of a lightweight three-dimensional graphene assembly. Sci. Adv..

[148] Butterfield B., Chapman K., Christie L., Dickson A. (1992). Ultrastructural characteristics of failure surfaces in medium density fibreboard. For. Prod. J..

[149] Carvalho L.M., Costa C.A. (1998). Modeling and simulation of the hot-pressing process in the production of medium density fiberboard (MDF). Chem. Eng. Commun..

[150] Travaglini S., Noble J., Ross P., Dharan C. Mycology matrix composites. Proceedings of the 28th Annual Technical Conference of the American Society for Composites (ASC).

[151] Elsacker E., Vandelook S., Damsin B., Van Wylick A., Peeters E., De Laet L. (2021). Mechanical characteristics of bacterial cellulose-reinforced mycelium composite materials. Fungal Biol. Biotechnol..

[152] Birinci A.U., Demir A., Ozturk H. (2022). Comparison of thermal performances of plywood shear walls produced with different thermal insulation materials. Maderas. Cienc. Tecnol..

[153] ASTM C. (2006). Standard Specification for Rigid Cellular Polystyrene Thermal Insulation.

[154] van Empelen J.C. (2018). A Study into More Sustainable, Alternative Building Materials as a Substitute for Concrete in Tropical Climates.

[155] Özlüsoylu İ., İstek A. The effect of hybrid resin usage on thermal conductivity in ecological insulation panel production. Proceedings of the 4th International Conference on Engineering Technology and Applied Sciences.

[156] Gibson L.J. (2012). The hierarchical structure and mechanics of plant materials. J. R. Soc. Interface.

[157] Yang L., Park D., Qin Z. (2021). Material function of mycelium-based bio-composite: A review. Front. Mater..

[158] Chan X.Y., Saeidi N., Javadian A., Hebel D.E., Gupta M. (2021). Mechanical properties of dense mycelium-bound composites under accelerated tropical weathering conditions. Sci. Rep..

[159] Tacer-Caba Z., Varis J.J., Lankinen P., Mikkonen K.S. (2020). Comparison of novel fungal mycelia strains and sustainable growth substrates to produce humidity-resistant biocomposites. Mater. Des..

[160] Mardijanti D.S., Megantara E.N., Bahtiar A., Sunardi S. (2021). Turning the cocopith waste into myceliated biocomposite to make an insulator. Int. J. Biomater..

[161] López Nava J., Méndez González J., Ruelas Chacón X., Nájera Luna J. (2016). Assessment of edible fungi and films bio-based material simulating expanded polystyrene. Mater. Manuf. Process..

[162] Spyridonos E., Witt M.-U., Dippon K., Milwich M., Gresser G.T., Dahy H. (2024). Natural fibre pultruded profiles: Illustration of optimisation processes to develop high-performance biocomposites for architectural and structural applications. Compos. Part C Open Access.

[163] Silverman J. (2018). Development and Testing of Mycelium-Based Composite Materials for Shoe Sole Applications.

[164] Alemu D., Tafesse M., Mondal A.K. (2022). Mycelium-based composite: The future sustainable biomaterial. Int. J. Biomater..

[165] Solomon A., Vinoth J., Sudhakar R., Hemalatha G. (2017). Inspecting the behavior of insulated concrete form (icf) blocks with polypropylene sheet. Indian J. Sci. Res.

[166] Wagner M., Biegler V., Wurm S., Baumann G., Nypelö T., Bismarck A., Feist F. (2025). Pulp fibre foams: Morphology and mechanical performance. Compos. Part A Appl. Sci. Manuf..

[167] Gao H., Liu J., Liu H. (2011). Geotechnical properties of EPS composite soil. Int. J. Geotech. Eng..

[168] Răut I., Călin M., Vuluga Z., Oancea F., Paceagiu J., Radu N., Doni M., Alexandrescu E., Purcar V., Gurban A.-M. (2021). Fungal based biopolymer composites for construction materials. Materials.

[169] Yang K. (2020). Investigations of Mycelium as a Low-Carbon Building Material. ENGS 88 Honors Thesis.

[170] Gou L., Li S., Yin J., Li T., Liu X. (2021). Morphological and physico-mechanical properties of mycelium biocomposites with natural reinforcement particles. Constr. Build. Mater..

[171] Dhillon G.S., Kaur S., Brar S.K., Verma M. (2013). Green synthesis approach: Extraction of chitosan from fungus mycelia. Crit. Rev. Biotechnol..

[172] Arifin Y.H., Yusuf Y. (2013). Mycelium fibers as new resource for environmental sustainability. Procedia Eng..

[173] Santos I.S., Nascimento B.L., Marino R.H., Sussuchi E.M., Matos M.P., Griza S. (2021). Influence of drying heat treatments on the mechanical behavior and physico-chemical properties of mycelial biocomposite. Compos. Part B Eng..

[174] Manan S., Ullah M.W., Ul-Islam M., Atta O.M., Yang G. (2021). Synthesis and applications of fungal mycelium-based advanced functional materials. J. Bioresour. Bioprod..

[175] Schritt H., Vidi S., Pleissner D. (2021). Spent mushroom substrate and sawdust to produce mycelium-based thermal insulation composites. J. Clean. Prod..

[176] Huang Z., Wei Y., Hadigheh S.A. (2024). Variations in the properties of engineered mycelium-bound composites (mbcs) under different manufacturing conditions. Buildings.

[177] Sratong-on P., Puttawongsakul K., Kantawee N. (2023). Physical and Mechanical Properties of Indian Oyster Mushroom Mycelium/Sawdust Composites for Biodegradable Packaging Materials. Curr. Appl. Sci. Technol..

[178] Jia N., Kagan V.A. (2001). Mechanical performance of polyamides with influence of moisture and temperature–accurate evaluation and better understanding. Plast. Fail. Anal. Prev..

[179] Li M.M., Pan H.C., Huang S.L., Scholz M. (2013). Controlled experimental study on removing diesel oil spillages using agricultural waste products. Chem. Eng. Technol..

[180] Wei L., Liang S., McDonald A.G. (2015). Thermophysical properties and biodegradation behavior of green composites made from polyhydroxybutyrate and potato peel waste fermentation residue. Ind. Crops Prod..

[181] Zabihzadeh S.M. (2010). Water uptake and flexural properties of natural filler/HDPE composites. BioResources.

[182] Mokhothu T.H., John M.J. (2017). Bio-based coatings for reducing water sorption in natural fibre reinforced composites. Sci. Rep..

[183] Corazzari I., Nisticò R., Turci F., Faga M.G., Franzoso F., Tabasso S., Magnacca G. (2015). Advanced physico-chemical characterization of chitosan by means of TGA coupled on-line with FTIR and GCMS: Thermal degradation and water adsorption capacity. Polym. Degrad. Stab..

[184] Pelletier M., Holt G., Wanjura J., Greetham L., McIntyre G., Bayer E., Kaplan-Bie J. (2019). Acoustic evaluation of mycological biopolymer, an all-natural closed cell foam alternative. Ind. Crops Prod..

[185] Sun W., Tajvidi M., Howell C., Hunt C.G. (2022). Insight into mycelium-lignocellulosic bio-composites: Essential factors and properties. Compos. Part A Appl. Sci. Manuf..

[186] Castagnede B., Aknine A., Brouard B., Tarnow V. (2000). Effects of compression on the sound absorption of fibrous materials. Appl. Acoust..

[187] Collet F., Pretot S. (2014). Thermal conductivity of hemp concretes: Variation with formulation, density and water content. Constr. Build. Mater..

[188] Schnabel T., Huber H., Petutschnigg A., Jäger A. (2019). Analysis of plant materials pre-treated by steam explosion technology for their usability as insulating materials. Agron. Res..

[189] Dias P.P., Jayasinghe L.B., Waldmann D. (2021). Investigation of Mycelium-Miscanthus composites as building insulation material. Results Mater..

[190] Pruteanu M. (2010). Investigations regarding the thermal conductivity of straw. Bul. Institutului Politeh. Iasi Sect. Constr. Arhit..

[191] Bergman T.L. (2011). Fundamentals of Heat and Mass Transfer.

[192] Das O., Mensah R.A., Balasubramanian K.B.N., Shanmugam V., Försth M., Hedenqvist M.S., Rantuch P., Martinka J., Jiang L., Xu Q. (2023). Functionalised biochar in biocomposites: The effect of fire retardants, bioplastics and processing methods. Compos. Part C Open Access.

[193] Jones M., Bhat T., Kandare E., Thomas A., Joseph P., Dekiwadia C., Yuen R., John S., Ma J., Wang C.-H. (2018). Thermal degradation and fire properties of fungal mycelium and mycelium-biomass composite materials. Sci. Rep..

[194] Teixeira J.L., Matos M.P., Nascimento B.L., Griza S., Holanda F.S.R., Marino R.H. (2018). Production and mechanical evaluation of biodegradable composites by white rot fungi. Ciência Agrotecnolog..

[195] Rantuch P., Kvorková V., Wachter I., Martinka J., Štefko T. (2024). Is biochar a suitable fire retardant for furfurylated wood?. Compos. Part C Open Access.

[196] Jin X., Cui S., Sun S., Gu X., Li H., Liu X., Tang W., Sun J., Bourbigot S., Zhang S. (2019). The preparation of a bio-polyelectrolytes based core-shell structure and its application in flame retardant polylactic acid composites. Compos. Part A Appl. Sci. Manuf..

[197] Chen N., Zhang S., Pan X., Zhou S., Zhao M. (2020). Foaming mechanism and optimal process conditions of foamed glass based on thermal analysis. J. Porous Mater..

[198] Agarwal G., Liu G., Lattimer B. (2014). Pyrolysis and oxidation of cardboard. Fire Saf. Sci..

[199] Houette T., Maurer C., Niewiarowski R., Gruber P. (2022). Growth and mechanical characterization of mycelium-based composites towards future bioremediation and food production in the material manufacturing cycle. Biomimetics.

[200] Aiduang W., Suwannarach N., Kumla J., Thamjaree W., Lumyong S. (2022). Valorization of agricultural waste to produce myco-composite materials from mushroom mycelia and their physical properties. Agric. Nat. Resour..

[201] Rigobello A., Ayres P. (2022). Compressive behaviour of anisotropic mycelium-based composites. Sci. Rep..

[202] Dizon J.R.C., Valino A.D., Souza L.R., Espera A.H., Chen Q., Advincula R.C. (2019). Three-dimensional-printed molds and materials for injection molding and rapid tooling applications. MRS Commun..

[203] Forest Products Laboratory (US), University of Wisconsin (1961). Manufacture and General Characteristics of Flat Plywood.

[204] Schroeder H.A. (1972). Shrinking and Swelling Differences Between Hardwoods and Softwoods.

[205] Rashidi L. (2021). Magnetic nanoparticles: Synthesis and characterization. Magnetic Nanoparticle-Based Hybrid Materials.

[206] Hu M., Cao X. (2025). Experimental Assessment of Multiple Properties of Mycelium-Based Composites with Sewage Sludge and Bagasse. Materials.

[207] Shen D., Gu S., Bridgwater A.V. (2010). Study on the pyrolytic behaviour of xylan-based hemicellulose using TG–FTIR and Py–GC–FTIR. J. Anal. Appl. Pyrolysis.

[208] Jiang L., Walczyk D., McIntyre G., Chan W.K. (2016). Cost modeling and optimization of a manufacturing system for mycelium-based biocomposite parts. J. Manuf. Syst..

[209] Osman E.Y. (2023). Economic Assessment of Mycelia-Based Composite in the Built Environment. Ph.D. Thesis.

[210] Ghoshal T., Parmar P.R., Bhuyan T., Bandyopadhyay D. (2023). Polystyrene foams: Materials, technology, and applications. Polymeric Foams: Fundamentals and Types of Foams (Volume 1).

[211] Barrera Castro G., Ocampo Carmona L., Olaya Florez J. (2017). Production and characterization of the mechanical and thermal properties of expanded polystyrene with recycled material. Ing. Y Univ..

[212] Monteiro S., de Assis F., Ferreira C., Simonassi N., Weber R., Oliveira M., Colorado H., Pereira A. (2018). Fique fabric: A promising reinforcement for polymer composites. Polymers.

[213] Vandi L.-J., Chan C.M., Werker A., Richardson D., Laycock B., Pratt S. (2018). Wood-PHA composites: Mapping opportunities. Polymers.

[214] Peeters S.S. (2023). Assessing Modifications on Mycelium-Based Composites and the Effects on Fungal Degradation and Material Properties.

[215] Indexbox. Cement Price per kg. https://www.indexbox.io/search/cement-price-per-kg/.

[216] Logan J., Buckley D. (1991). Subterranean termite control in buildings. The Chemistry of Wood Preservation.

[217] Guillebeau L.P., Hinkle N., Roberts P. (2008). Summary of Losses from Insect Damage and Cost of Control in Georgia 2006.

[218] Bajwa D.S., Holt G.A., Bajwa S.G., Duke S.E., McIntyre G. (2017). Enhancement of termite (*Reticulitermes flavipes* L.) resistance in mycelium reinforced biofiber-composites. Ind. Crops Prod..

[219] Vachon J., Assad-Alkhateb D., Baumberger S., Van Haveren J., Gosselink R.J., Monedero M., Bermudez J.M. (2020). Use of lignin as additive in polyethylene for food protection: Insect repelling effect of an ethyl acetate phenolic extract. Compos. Part C Open Access.

[220] Bultman J., Chen S.-L., Schloman W. (1998). Anti-termitic efficacy of the resin and rubber in fractionator overheads from a guayule extraction process. Ind. Crops Prod..

[221] Zhu B.C., Henderson G., Chen F., Fei H., Laine R.A. (2001). Evaluation of vetiver oil and seven insect-active essential oils against the Formosan subterranean termite. J. Chem. Ecol..

[222] Abrams M. (2014). Construction Materials Made from ‘Shrooms.

[223] Volk R., Schröter M., Saeidi N., Steffl S., Javadian A., Hebel D.E., Schultmann F. (2024). Life cycle assessment of mycelium-based composite materials. Resour. Conserv. Recycl..

[224] Guinée J.B. (2002). Handbook on Life Cycle Assessment: Operational Guide to the ISO Standards.

[225] Stelzer L., Hoberg F., Bach V., Schmidt B., Pfeiffer S., Meyer V., Finkbeiner M. (2021). Life cycle assessment of fungal-based composite bricks. Sustainability.

[226] Ravichandran B., Balasubramanian M. (2024). Joining methods for Fiber Reinforced Polymer (FRP) composites—A critical review. Compos. Part A Appl. Sci. Manuf..

[227] Alaux N., Vašatko H., Maierhofer D., Saade M.R.M., Stavric M., Passer A. (2024). Environmental potential of fungal insulation: A prospective life cycle assessment of mycelium-based composites. Int. J. Life Cycle Assess..

[228] Potrč Obrecht T., Jordan S., Legat A., Passer A. (2021). The role of electricity mix and production efficiency improvements on greenhouse gas (GHG) emissions of building components and future refurbishment measures. Int. J. Life Cycle Assess..

[229] Zhang X. (2022). The influence of future electricity supplies in life cycle assessment (LCA) of buildings. IEA EBC Annex.

[230] McNeill D.C., Pal A.K., Nath D., Rodriguez-Uribe A., Mohanty A.K., Pilla S., Gregori S., Dick P., Misra M. (2024). Upcycling of Ligno-Cellulosic Nutshells Waste Biomass in Biodegradable Plastic-based Biocomposites Uses—A Comprehensive Review. Compos. Part C Open Access.

[231] Gosden E. Ikea Plans Mushroom-Based Packaging as Eco-Friendly Replacement for Polystyrene. The Telegraph. 24 February 2016. https://www.telegraph.co.uk/news/earth/businessandecology/recycling/12172439/Ikea-plans-mushroom-based-packaging-as-eco-friendly-replacement-for-polystyrene.html.

[232] Asdrubali F., D’Alessandro F., Schiavoni S. (2015). A review of unconventional sustainable building insulation materials. Sustain. Mater. Technol..

[233] Dicker M.P., Duckworth P.F., Baker A.B., Francois G., Hazzard M.K., Weaver P.M. (2014). Green composites: A review of material attributes and complementary applications. Compos. Part A Appl. Sci. Manuf..

[234] Jones M., Bhat T., Wang C.H., Moinuddin K., John S. Thermal degradation and fire reaction properties of mycelium composites. Proceedings of the 21st International Conference on Composite Materials.

[235] Ecovative Design L. (2019). Mycocomposite—Mycelium-Bound Agricultural Byproducts. http://ecovativedesign.com/mycocomposite.

[236] Sydor M., Bonenberg A., Doczekalska B., Cofta G. (2021). Mycelium-based composites in art, architecture, and interior design: A review. Polymers.

[237] Design K. (2021). Beautiful Products with Fungus and Biomass. https://www.mycote.ch/mycotree.

[238] Zamani A. (2010). Superabsorbent Polymers from the Cell Wall of Zygomycetes Fungi.

[239] Vasquez E.S.L., Vega K. Myco-accessories: Sustainable wearables with biodegradable materials. Proceedings of the 2019 ACM International Symposium on Wearable Computers.

[240] Edebo L. (2002). Porous Structure Comprising Fungi Cell Walls. U.S. Patent.

[241] Saini R., Kaur G., Brar S.K. (2023). Textile residue-based mycelium biocomposites from *Pleurotus ostreatus*. Mycology.

[242] Janesch J., Jones M., Bacher M., Kontturi E., Bismarck A., Mautner A. (2020). Mushroom-derived chitosan-glucan nanopaper filters for the treatment of water. React. Funct. Polym..

[243] Zhao A., Berglund L., Rosenstock Völtz L., Swamy R., Antonopoulou I., Xiong S., Mouzon J., Bismarck A., Oksman K. (2025). Fungal Innovation: Harnessing Mushrooms for Production of Sustainable Functional Materials. Adv. Funct. Mater..

[244] Oksman K., Aitomäki Y., Mathew A.P., Siqueira G., Zhou Q., Butylina S., Tanpichai S., Zhou X., Hooshmand S. (2016). Review of the recent developments in cellulose nanocomposite processing. Compos. Part A Appl. Sci. Manuf..

[245] Zhan M., Wool R.P. (2013). Design and evaluation of bio-based composites for printed circuit board application. Compos. Part A Appl. Sci. Manuf..

[246] Vasquez E.S.L., Vega K. (2019). From plastic to biomaterials: Prototyping DIY electronics with mycelium. Adjunct Proceedings of the 2019 ACM International Joint Conference on Pervasive and Ubiquitous Computing and Proceedings of the 2019 ACM International Symposium on Wearable Computers, London, UK, 9–13 September 2019.

[247] Heide A., Wiebe P., Sabantina L., Ehrmann A. (2023). Suitability of Mycelium-Reinforced Nanofiber Mats for Filtration of Different Dyes. Polymers.

[248] Soon C.F., Yee S.K., Nordin A.N., Rahim R.A., Ma N.L., Hamed I.S.L.A., Tee K.S., Azmi N.H., Sunar N.M., Heng C. (2024). Advancements in Biodegradable Printed Circuit Boards: Review of Material Properties, Fabrication Methods, Applications and Challenges. Int. J. Precis. Eng. Manuf..

[249] Rapagnani N., van Bezooijen A., Borruso L., Mimmo T., Bouaicha O. (2024). Bio Design for Footwear Innovation: Growing Sneaker Components with Composite Mycelium-Based Materials.

[250] Oliver-Ortega H., Geng S., Espinach F.X., Oksman K., Vilaseca F. (2021). Bacterial cellulose network from kombucha fermentation impregnated with emulsion-polymerized poly (methyl methacrylate) to form nanocomposite. Polymers.

[251] Lee K.-Y., Aitomäki Y., Berglund L.A., Oksman K., Bismarck A. (2014). On the use of nanocellulose as reinforcement in polymer matrix composites. Compos. Sci. Technol..

[252] Bakare F.O., Ramamoorthy S.K., Åkesson D., Skrifvars M. (2016). Thermomechanical properties of bio-based composites made from a lactic acid thermoset resin and flax and flax/basalt fibre reinforcements. Compos. Part A Appl. Sci. Manuf..

[253] Hietala M., Mathew A.P., Oksman K. (2013). Bionanocomposites of thermoplastic starch and cellulose nanofibers manufactured using twin-screw extrusion. Eur. Polym. J..

[254] Früchtl M., Senz A., Sydow S., Frank J.B., Hohmann A., Albrecht S., Fischer M., Holland M., Wilhelm F., Christ H.-A. (2023). Sustainable pultruded sandwich profiles with mycelium core. Polymers.

[255] Jonoobi M., Harun J., Mathew A.P., Oksman K. (2010). Mechanical properties of cellulose nanofiber (CNF) reinforced polylactic acid (PLA) prepared by twin screw extrusion. Compos. Sci. Technol..

[256] Simard S.W., Beiler K.J., Bingham M.A., Deslippe J.R., Philip L.J., Teste F.P. (2012). Mycorrhizal networks: Mechanisms, ecology and modelling. Fungal Biol. Rev..

[257] Gorzelak M.A., Asay A.K., Pickles B.J., Simard S.W. (2015). Inter-plant communication through mycorrhizal networks mediates complex adaptive behaviour in plant communities. AoB Plants.

[258] Fricker M.D., Heaton L.L., Jones N.S., Boddy L. (2017). The mycelium as a network. Fungal Kingd..

[259] Al-Taweil H.I., Osman M.B., Abdulhamid A., Mohammad N., Wan Yussof W.M. (2010). Microbial inoculants for enhancing rice growth and sheath spots disease suppression. Arch. Agron. Soil Sci..

[260] Elnahal A.S., El-Saadony M.T., Saad A.M., Desoky E.-S.M., El-Tahan A.M., Rady M.M., AbuQamar S.F., El-Tarabily K.A. (2022). The use of microbial inoculants for biological control, plant growth promotion, and sustainable agriculture: A review. Eur. J. Plant Pathol..

[261] Vassilev N., Mendes G.d.O. (2024). Soil Fungi in Sustainable Agriculture. Microorganisms.

[262] Gianinazzi S., Gollotte A., Binet M.-N., van Tuinen D., Redecker D., Wipf D. (2010). Agroecology: The key role of arbuscular mycorrhizas in ecosystem services. Mycorrhiza.

